# Impact of rising seawater temperature on a phagocytic cell population during *V. parahaemolyticus* infection in the sea anemone *E. pallida*

**DOI:** 10.3389/fimmu.2023.1292410

**Published:** 2023-11-22

**Authors:** Mélanie Billaud, Frédéric Larbret, Dorota Czerucka

**Affiliations:** ^1^ Biomedical Department, Scientific Center of Monaco, Monaco, Monaco; ^2^ LIA ROPSE, Laboratoire International Associé, Centre Scientifique de Monaco, Université Côte d’Azur, Nice, France; ^3^ Université Côte d’Azur, L’Institut national de la santé et de la recherche médicale (INSERM), Centre Méditerranéen de Médecine Moléculaire (C3M), Nice, France

**Keywords:** *Exaiptasia pallida*, climate changes, *Vibrio parahaemolyticus*, phagocytosis, spectral flow cytometry, autofluorescence, innate immunity, amebocyte-like cell

## Abstract

Climate change is increasing ocean temperatures and consequently impacts marine life (e.g., bacterial communities). In this context, studying host–pathogen interactions in marine organisms is becoming increasingly important, not only for ecological conservation, but also to reduce economic loss due to mass mortalities in cultured species. In this study, we used *Exaiptasia pallida* (*E. pallida*), an anemone, as an emerging marine model to better understand the effect of rising temperatures on the infection induced by the pathogenic marine bacterium *Vibrio parahaemolyticus*. The effect of temperature on *E. pallida* was examined at 6, 24, or 30 h after bath inoculation with 10^8^ CFU of *V. parahaemolyticus* expressing GFP (Vp-GFP) at 27°C (husbandry temperature) or 31°C (heat stress). Morphological observations of *E. pallida* and their Hsps expression demonstrated heat stress induced increasing damage to anemones. The kinetics of the infections revealed that Vp-GFP were localized on the surface of the ectoderm and in the mucus during the first hours of infection and in the mesenterial filaments thereafter. To better identify the *E. pallida* cells targeted by Vp-GFP infection, we used spectral flow cytometry. *E. pallida* cell types were identified based on their autofluorescent properties. corresponding to different cell types (algae and cnidocytes). We identified an AF10 population whose autofluorescent spectrum was identical to that of human monocytes/macrophage, suggesting that this spectral print could be the hallmark of phagocytic cells called “amebocytes’’. AF10 autofluorescent cells had a high capacity to phagocytize Vp-GFP, suggesting their possible role in fighting infection. This was confirmed by microscopy using sorted AF10 and GFP-positive cells (AF10+/GFP+). The number of AF10+/GFP+ cells were reduced at 31°C, demonstrating that increased temperature not only damages tissue but also affects the immune response of *E. pallida*. In conclusion, our study provides a springboard for more comprehensive studies of immune defense in marine organisms and paves the way for future studies of the dynamics, activation patterns, and functional responses of immune cells when encountering pathogens.

## Introduction

The Earth’s climate is undergoing rapid changes due to global warming. The Intergovernmental Panel on Climate Change (IPCC) has clearly documented the trend of rising ocean temperatures and attributed this rise to the accumulation of greenhouse gases in the Earth’s atmosphere. IPCC estimates that the average global sea surface temperature will rise from 27°C to 31°C in 2050 and projections indicate a continuation of this alarming trend in the 21st century (1).

Indeed, studies have shown an alarming increase in the presence of *Vibrio* spp. (i.e., *V. cholerae*, *V. vulnificus*, *V. alginolyticus*, and *V. parahaemolyticus)* in the oceans, which not only pose significant challenges to the health and survival of marine organisms (2–7) but also are known to cause infections in humans. We have previously demonstrated that an increase in seawater temperature (SWT) from 27°C to 31°C, upregulated the expression by *V. parahaemolyticus* of virulence factors responsible for adhesion and biofilm formation on biotic and abiotic surfaces, thereby promoting bacterial spread and virulence (8).

Majority of actual research focuses on the impact of pathogens and temperature shift on cnidarians; however, these studies examined heterotrophy (9), decalcification (10), symbiont exchange (11), and inducible secondary metabolites (12) as mechanisms by which corals can overcome changing ocean conditions. There are no studies to date that have directly studied host immunity and plasticity of innate immunity in field conditions. This gap in research highlights the need for investigations into the immune responses of key marine organisms when confronted with temperature-induced stress as a result of an infection.

Among cnidarians, the symbiotic sea anemone *Exaiptasia pallida* (*E. pallida*) is an emerging model used for studying host–pathogen interactions, underlying immune responses and potential acclimation strategies (13–16). While natural corals are becoming less available for laboratory research, *E. pallida* offers the advantage of being able to be cultured in large quantities. In addition, *E. pallida* is an anthozoan that has an open circulatory system that also forms symbiotic associations with dinoflagellates but lacks the characteristic calcium carbonate skeleton of corals (16). Moreover, the availability of clonal populations of *E. pallida* with identical genetic backgrounds facilitates genetic investigations, a clear advantage for studying potential acclimatization processes. Although recently the generation and analysis of the transcriptome of *E. pallida* has been described (13, 17), our understanding of the interactions between this model polyp and opportunistic coral pathogens remains limited.

In this paper, we investigated the effect of rising SWTs (27°C vs 31°C) on *E. pallida* immune responses to the infection induced by *V. parahaemolyticus.* A previous study from our group has shown that *E. pallida* overexpressed many genes involved in the innate immune response to *V. parahaemolyticus*-induced infection, including mucus secretion, production of antimicrobial peptides, inflammation, and apoptotic pathway (18). We have also investigated the dissemination of *V. parahaemolyticus* in the whole animal and identified different types of mucus cells (protective cells), cnidocytes (involved in defense and nutrition), algae (photosynthesis), and bacteria clustered in cells (Billaud et al., in review).

Engulfment of pathogenic bacteria by phagocytic cells such as monocytes or macrophages is the first innate immune response to infection in vertebrates (e.g., humans) (19). Phagocytic cells called “amebocytes” have been identified in many marine organisms (i.e., echinoderm and cnidarian) (20–23). Amebocytes are critical for immune defense by recognizing and eliminating harmful substances and pathogens. In *Swiftia exserta*, these cells were observed to become active during phagocytosis when foreign particles were introduced (24). Recently, cells exhibiting high phagocytic activity have also been characterized by traditional flow cytometry (FACS) in corals and anemones (21, 25). In that study, phagocytes have been identified based on their granularity and size, and engulfment of fluorescent beads or bacterial particles.

Here, we used the cell’s autofluorescence properties to characterize different cell types in *E. pallida* by spectral flow cytometry. Spectral flow cytometry offers several advantages due to its ability to resolve complex mixtures of fluorescence spectra. Cells have a natural level of fluorescence, called autofluorescence (AF) due to the presence of inherent fluorescent components such as collagen and elastin, cyclic ring compounds like NADPH and riboflavin, aromatic amino acids, and cellular organelles such as mitochondria and lysosomes. Autofluorescence has already been used in spectral flow cytometry to characterize fetal liver stromal cells or white blood cells (26, 27).

Using this approach, we identify different cell types in the sea anemone *E. pallida* (Algae, Cnidocyte). We have also characterized a specific print spectrum of an “amebocyte-like cell” population (AF10) that has interestingly shown similarity to human monocytes/macrophages. This population, which can phagocytes Vp-GFP, was mainly found in the mesenterial filaments of *E. pallida* infected at normal temperature (27°C) but was reduced in elevated temperatures (31°C). Together, these data suggest that AF10 cells are implicated in the immune response of *E. pallida* and have a temperature-dependent activity.

## Materials and methods

### Bacterial strain

*Vibrio parahaemolyticus* RIMD2210633 strain serotype O3:K6 expressing GFP (Vp-GFP) was provided by Kim Orth from UT Southwestern, Dallas, Texas (28). The strain was maintained at −80°C in a 25% glycerol solution. To thaw the bacteria, Vp-GFP is inoculated into 5 mL of Luria-Bertani medium (LB) containing 3% NaCl supplemented with 50 µg/mL of spectinomycin (standard condition) for 6 h.

### Animal husbandry at 27 and 31°C

*Exaiptasia pallida (E. pallida)* strains CC7 were acquired from the Pringle laboratory at Stanford University. Sea anemones were maintained in 1-L tanks incubated in 0.22 µm of filtered seawater (FSW) at 27°C or 31°C with a 12-h/12-h light–dark cycle. Feeding was done following the Pringle Lab feeding anemone protocol (https://dx.doi.org/10.17504/protocols.io.rkud4ww). To study the infectious process at 31°C and to overcome the heat shock effect, we maintained anemones for at least 2 weeks at 31°C before infection. FSW was changed and tanks were cleaned twice per week with cell scrapers and pipettes. If any anemones were dying, they were removed from the tanks. The anemones were fed with artemia twice a week. They were starved only 1 week before the experiment to remove the effect of artemia feeding.

### *E. pallida* infection with *V. parahaemolyticus*-GFP

Prior to exposure, six anemones per condition with an approximate diameter of half a centimeter (tentacles and pedal relaxed) were transferred from stock tanks into the 12-well plates with 2 mL of filter-sterilized (0.22 μm) seawater (FSW) supplemented with 50 µg/mL of spectinomycin. Anemones were kept still overnight until fixed to the bottom of the plate and acclimatization.

Vp-GFP solution was subcultured overnight in LB medium supplemented with NaCl 3% and spectinomycin 50 μg/mL (37°C). Cultures were harvested by centrifugation (2,500 × *g*, 15 min). Bacterial cultures were resuspended in 1 mL of 0.22 µm FSW supplemented with 50 μg/mL spectinomycin to an OD620 nm ~0.8 corresponding to 10^9^ CFU/mL. The final concentration for infection was 2.5 × 10^8^ CFU/well (in 2 mL). After 6, 24, or 30 h, the anemones were relaxed with 7.14% of MgCl_2_ in FSW for 10 min and fixed overnight in PFA 4% in FSW or kept alive.

### RNA extraction

Following each time point (*n* = 6 minimum), alive *E. pallida* anemones were placed separately in a 2-mL tube and incubated for 5 min at RT with 1 ml of Trizol Reagent (Invitrogen). All samples were homogenized with Zirconium beads using a Precellys Homogenizer (2,600 revolutions 2× 30 s at RT). RNA was purified using DirectZol kit (ZymoResearch) following the manufacturer’s instructions. Total RNA was quantified at 260-nm wavelength using a Synergy H1M spectrophotometer.

### Reverse transcription

*E. pallida* RNA was used for the determination of the expression of stress factors. Complementary DNA (cDNA) was constructed using the RevertAid First Strand cDNA Synthesis Kit (Fermentas/Thermoscientific, K1622) according to the manufacturer’s instructions.

### qPCR

The quantitative polymerase chain reaction (qPCR) was performed using the Applied Biosystems Real-Time PCR instrument. Fifteen nanograms of cDNA was placed in triplicate in each well of the qPCR plate, and a solution was added containing: 10 µM of forward primer, 10 µM of reverse primer, 0.1 µl of carboxy-X-rhodamine, 4.9 µl of RNase-free water, and 10 µl of SYBR Green, to obtain a final volume of 20 µl per well. The primers used are listed in **Supplementary Table 1**. The plate is then briefly centrifuged. Efficiencies of the primers were in the 95%–105% range. Quantification was determined using the comparative cycle threshold (CT) method relative to housekeeping gene RPL11, which is a ribosomal component. Expression of HSP40 and HSP70 genes was measured at baseline and following stress conditions. The average expression obtained of anemone controls was normalized to 1 for relative quantification expression (RQ). Experiment was performed with at least six anemones per condition.

### AnemoClear clearing technique

AnemoClear technique was optimized to clear *E. pallida* to make it transparent (removing lipids and pigments) to analyze the location of the fluorescent bacteria (Vp-GFP) in the whole organism. This technique is innovative because it allows us to visualize in 3D the whole anemone, localize bacteria inside the different organs, and maintain integrity of the tissues without blade strike. Controls and infected anemones were fixed in PFA 4% for 1 h at RT (*n* = 3). Clearing of anemones was performed using the AnemoClear protocol (Billaud et al., submitted). Acquisition was done under the Confocal Spinning W1 and 3D analysis with the Imaris Software.

### Fluorescent staining and immunohistochemistry

Fixed anemones were placed vertically into a mounting cup (Simport Scientific) and were frozen in Cryomatrix Frozen Embedding Medium (Thermo Scientific™ Shandon). Transversal sections (10 µm thick) were cut using a cryostat (Leica CM350). Slides were washed twice with 300 µL of PBS + Tween 0.1% and incubated with 300 µL of 0.1% Triton X-100 for 20 min to permeabilize the cells. After incubation, slides were washed twice with PBS Tween 0.1% before the blocking step with 300 µL of bovine serum albumin (BSA) 1% diluted in PBS Tween for 1 h. Cell membranes were stained with BioTracker Alexa 655 (Sigma-Aldrich Ref SCT108) for 30 min. Gland cells containing mucus were labeled with wheat germ agglutinin (WGA) conjugated with Texas Red (Invitrogen™ Ref W21405) following incubation for 30 min. For DNA labeling, 4′,6-diamidino-2-phenylindole (DAPI), was used (Invitrogen™ Ref D21490). After washing twice in PBS Tween 0.1%, slides were mounted using a small drop of Mowiol mounting medium and observed under a Nikon A1R Eclipse Ti2 microscope. ImageJ software was used to process and analyze the acquired images.

### Phagocytosis and digestion assays

For phagocytic and digestion tests, six anemones per condition with a diameter of about half a centimeter (tentacles and pedal relaxed) were transferred from the stock tanks into the 12-well plates with 2 mL of FSW supplemented with 50 µg/mL of spectinomycin. Anemones were kept still overnight until fixed to the bottom of the plate and acclimatization.

The pHrodo™ Green Zymosan Bioparticles (Thermo Fisher Scientific) were used for the *in vivo* phagocytosis assay. Bioparticles, which are a yeast antigen, were added into each well at 20 µg/ml. The fluorescence was bright only upon vesicle fusion with low pH vesicles, indicating the creation of phagolysosomes.

For the digestion test, 20 µg/ml of DQ™ Red BSA was used as a fluorogenic substrate for proteases (Invitrogen™, Ref D12051). The molecular Probe DQ Red BSA is a derivative of BSA that is labeled with BODIPY TR-X. The dyes were strongly self-quenched. The digestion assay protocol evaluates the proteolysis of the BSA conjugates by monitoring the fluorescence emitted by the protein fragments that contain isolated fluorophores.

The experiment was conducted over 24 h. Fluorescence microscopy was used on living animals to evaluate the location of phagocytosis activity with the pHrodo™ Green zymosan and the digestion of DQ™ BSA by measuring the overall fluorescence.

### Identification of *E. pallida* cell populations by spectral cytometry

Fixed anemones were dissected under a stereomicroscope into four tissues: body, tentacles, mesenteries, and acontia. Mesenteries and acontia are located in the coelenteron cavity of anemones. The dissection was done with very fine point tweezers inside a 20-mL petri dish filled with 10 mL of PBS Tween 0.1%. Dissected tissues were mechanically dissociated by filtration through 40-µm cell strainers with PBS. A syringe plunger was used to help facilitate the filtering. Cellular homogenates were analyzed with SpectroFlow software (version 3.1.0) on an Aurora spectral cytometer Cell sorter (Cytek) equipped with four lasers (405 nm, 488 nm, 560 nm, and 630 nm). Several N×N plots were done to separate the population depending on the 54 different channels. Different spectra of non-infected *E. pallida* cell populations (algae, cnidocytes and AF10) were identified. Debris and isolated bacteria were removed by a gate “cell”. Red channel was used to separate the algae from the other populations, especially the R7 channel, which corresponds to the highest intensity detected in the Algae spectrum. Cnidocytes were detected based on their large size in FSC. AF10+ cells were isolated in the V7 (violet laser)/B3 (blue laser) channels.

### Identification of amebocyte-like cells by spectral cytometry during Vp-GFP infection

For identification of infected “amebocyte-like cells”, Vp-GFP spectrum was identified and used as a fluorescence tag. Percentage of GFP-positive cells was computed from “not algae events” (which disturbs our analysis due to their high autofluorescence in each channel). Dual positive events AF10+/GFP+ (Autofluorescence population 10 “AF10” were considered as “amebocyte like cell” because of their final dual positivity with GFP) were sorted into 1.5-mL Eppendorf tubes with 300 µL of PBS. Sorted cells were labeled with DAPI (Invitrogen™ Ref D21490) and BioTracker Alexa 655 (Sigma-Aldrich Ref SCT108) following incubation of 30 min. After incubation, cells were centrifuged for 20 min at 30 × *g* at RT, resuspended in 30 µL of PBS, and plated into an 18 flat Well µ-Slide (Ibidi Ref 81826). Cells were observed under a Nikon A1R Eclipse Ti2 microscope. ImageJ software was used to process and analyze our acquisitions.

### Human monocyte culture

Human peripheral blood was obtained from healthy donors with informed consent following the Declaration of Helsinki. The project has been validated by The Etablissement Français du Sang and the French national agency for blood collection (79-52-NQ). Blood samples were collected using ethylene diamine tetra acetic acid-containing tubes. Mononucleated cells were first isolated by Ficoll Hypaque (Eurobio) and then enriched using autoMACS*Pro Separator (Miltenyi, France).

Spectral flow cytometry was used to identify specific autofluorescence spectra for different populations of human white blood cells. These spectra were distinguished based on three different morphological gates. Each gate was characterized by a five-color staining panel consisting of CD19 cFluor^®^R685, CD3 BV570, CD56 BV750, CD14 cFluor^®^B548, and CD16 cFluor^®^BYG610 obtained from CYTEK^®^’s 25- COLOR IMMUNOPROFILING ASSAY (reference: R7-40002). Cytometry acquisition was performed after fixation in PFA 4%, as previously described for *E. pallida* cells.

### Statistical analysis

The results are expressed as the mean ± standard error of mean (SD). Statistical studies were performed by analysis of means (*t*-test). The *p*-values < 0.05 were considered as statistically significant.

## Results

### Stress response of *E. pallida* during infection by *V. parahaemolyticus* depends on the temperature

**Figure 1:**
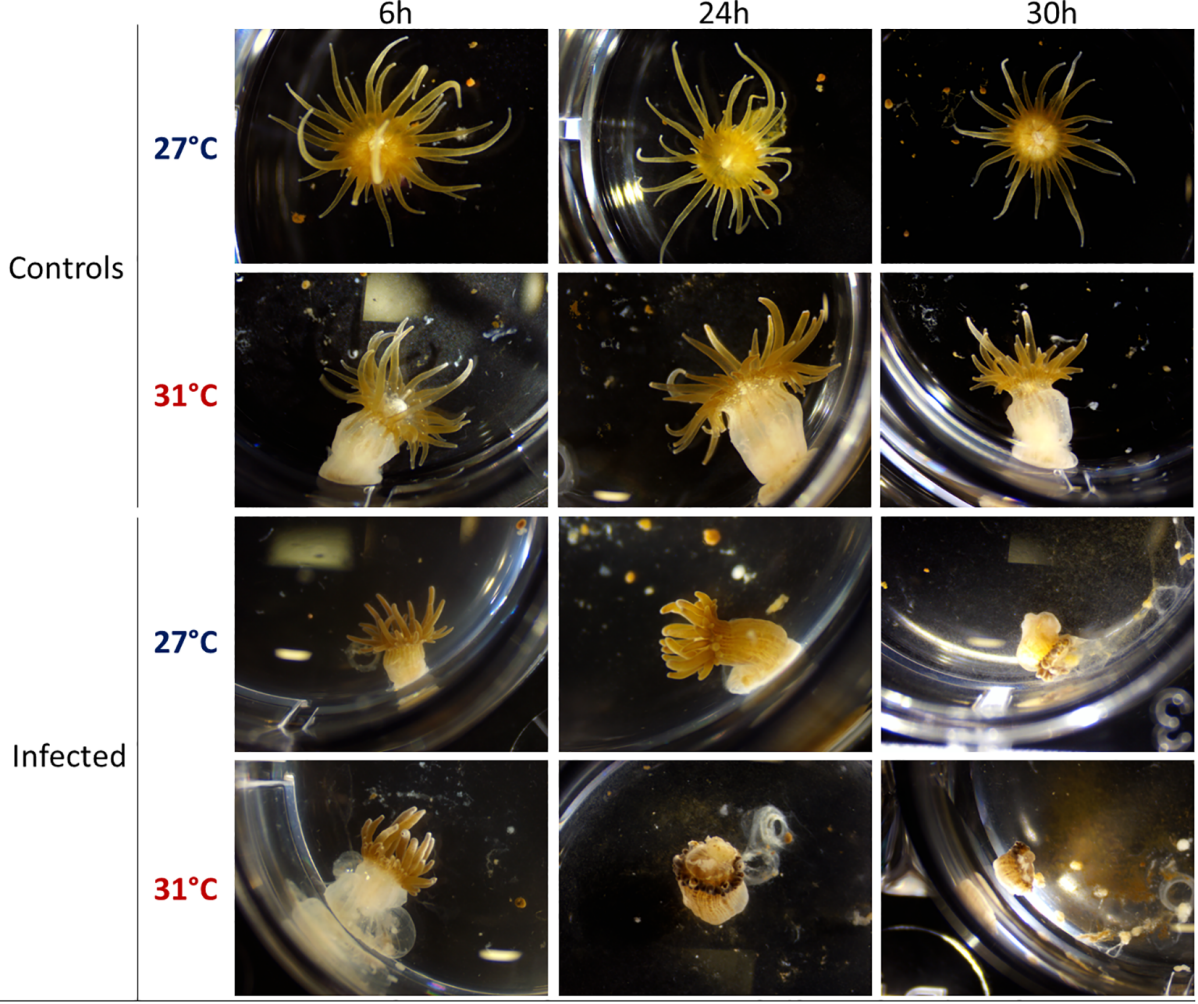
Binocular observation (x0.8) of *E. pallida* controls and infected by Vp-GFP after 6, 24, or 30 h in filtered sea water at 27°C (blue) or 31°C (red).

**Figure 2:**
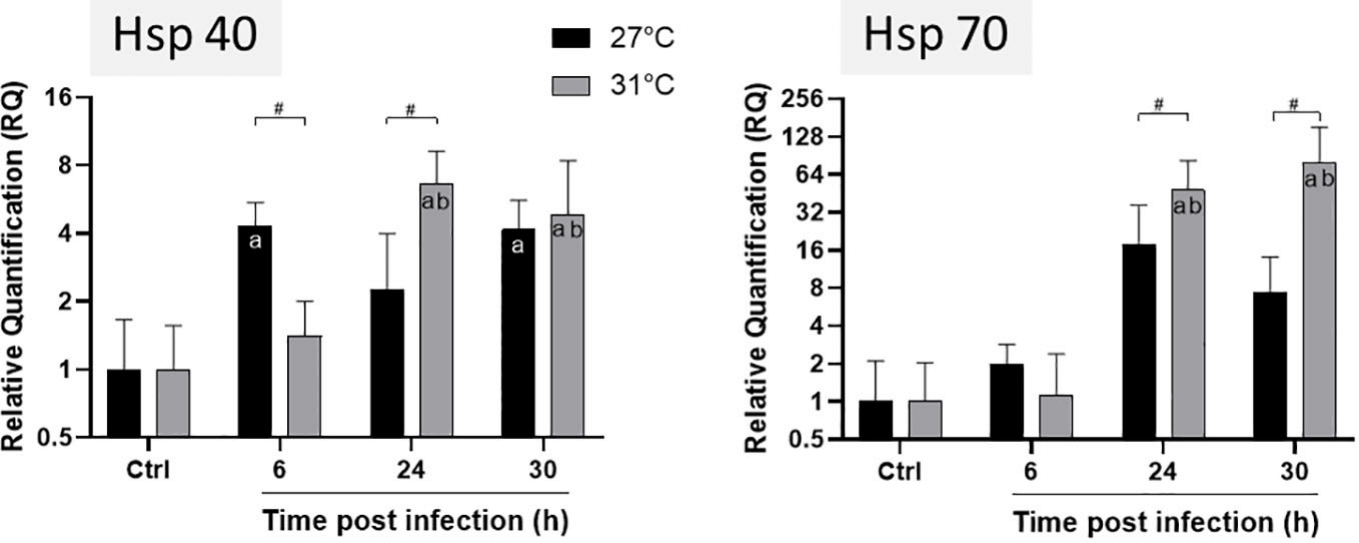
Expression levels of HSP40 and HSP70 genes of *E. pallida* at different time points. Infection was conducted at 27°C (black) and 31°C (grey) and samples were taken at 6, 24 and 30 h post infection before RNA extraction. Relative quantification (RQ) expressions are expressed as means ± standard deviations of results from experiments performed with six replicate samples. Statistics legends: ^#^Significant difference between 27 and 31°C. a. Significant difference compared to the control at the same temperature. b. Significant difference with 6 hours of infection at 31°C.

*E. pallida* is usually maintained at 27°C. Increase of temperature from 27°C to 31°C induces a stress response in *E. pallida* that can be followed by morphological modification and expression of heat shock proteins (Hsps). To study the infectious process at 31°C and to overcome the heat shock effect, we maintained anemones for at least 2 weeks at 31°C before infection. However, in anemones maintained at 31°C, we observed a retraction of tentacles when compared to animals maintained at 27°C (**Figure 1**). We verified that after this period of acclimatization, the level of Hsp 40/70 expression was not affected when compared to anemone maintained at 27°C (**Figure 2**). Amplified morphological changes were observed over time in infected animals. Morphological modifications appeared more rapidly in animals infected at 31°C when compared to infection at 27°C (**Figure 1**). After 24 h of infection with Vp, anemones at 31°C produced an external mucus ring to trap the bacteria Vp-GFP inside (see below), began to excrete parts of the mesenterial filaments and algae, and retracted their tentacles. Thirty hours post infection, the anemones were subject to greater damage at 31°C compared to damage seen at 27°C.

We followed Hsp gene expressions in animals infected at different temperatures (**Figure 2**). HSP40 reached its maximum expression at 6 h post infection at 27°C whereas at 31°C, maximum was reached after 24 h (**Figure 2**). The expression of HSP70 gene tends to increase not significantly at 27°C during infection, whereas at 31°C, it increased significantly after 24 h of infection and remained constant until 30 h. We observed a significant difference in the level of Hsp expression at the different temperatures: both HSP40 and HSP70 were significantly amplified from 24 to 30 h post infection at 31°C compared to 27°C. These results suggest that anemones are more stressed and susceptible to infection at 31°C than at 27°C.

### *V. parahaemolyticus* are mainly located in the mesenterial filaments of *E. pallida*

For *V. parahaemolyticus* localization, we performed a clearing protocol that we optimized for *E. pallida* anemones. This “AnemoClear” technique allowed us to explore not only the structure of *E. pallida* tissues under physiological conditions but also the interaction between anemones and the potential pathogens (Billaud et al., submitted).

**Figure 3:**
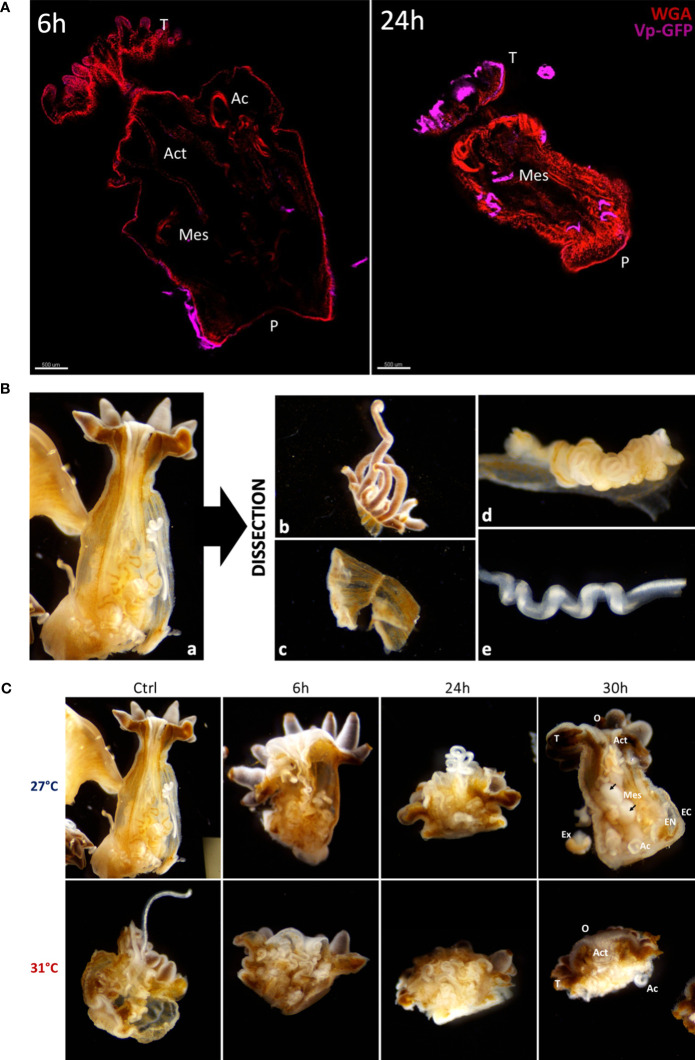
**(A)** Confocal microscopy obtained from cleared anemones thanks to the AnemoClear method. On the left: *E. pallida* anemone infected for 6 hours and on the right: *E. pallida* during 24 h at 27°C. Anemones were labelled with WGA for mucus cells (red) and with GFP antibodies for Vp-GFP bacteria (pink). Act Actinopharynx; Ac Acontia; Mes Mesenterial filaments; P Pedal disk; T Tentacles. Scale bar = 500 µm. **(B)** Binocular observation (×4) of longitudinal dissection of an anemone with all organs (a). Post dissection organs separated in 4 groups: (b) Tentacles; (c) Body; (d) Mesenterial filaments and (e) Acontia. **(C)** Binocular observation (×4) of longitudinal dissection of anemones controls and infected by Vp-GFP after 6, 24 and 30 hours at 27°C or 31°C post fixation. O Oral disk; T Tentacles; Act Actinopharynx; Mes Mesenterial filaments; EC Ectoderm; EN Endoderm; Ac Acontia; Ex Excreta. At 27°C after 30h of infection, round mesenteries are found (black arrows).

By using this method, we observed that tissue location of Vp-GFP in anemones infected at 27°C was changing over the time of infection. After 6 h of infection, the bacteria were mostly located on the ectoderm (external tissue of anemones), while after 24 h, they appeared in mesenterial filaments (**Figure 3A**).

To further monitor the infection process across the various tissues of anemones at 27°C or at 31°C, anemones were dissected at different time points of infection (6, 24, and 30 h). The whole anemones (a) were dissected into four tissues: (b) tentacles, (c) body, (d) mesenterial filaments, and (e) acontia (**Figure 3B**). We observed that all tissues were less damaged by the infection at 27°C than infection at 31°C (**Figure 3C**). The visual degradation seems to start earlier at 31°C after 24 h of infection with tentacle retractation, acontia, and algae expulsion and organ excretion compared to degradation seen at 27°C.

**Figure 4:**
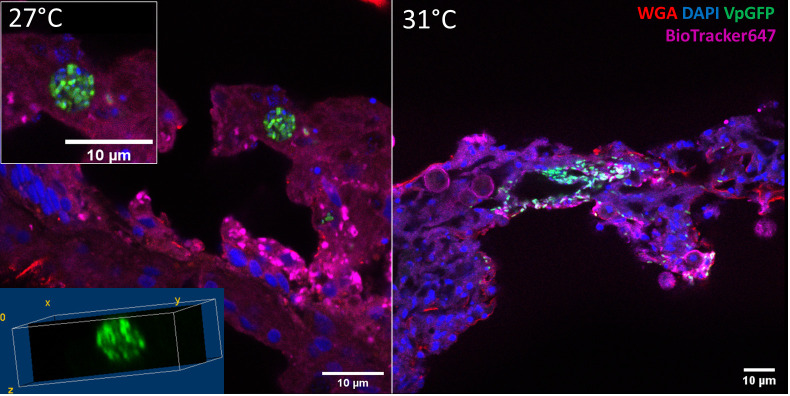
Confocal microscopy of a section of infected *E. pallida* by Vp-GFP at 27°C (left) or 31°C (right). Nuclei were labeled by DAPI (blue); mucus was labeled by WGA (red); Vp express constitutively GFP (green) and membrane by BioTracker655 (pink).

To determine the position of Vp-GFP in tissues more precisely, we performed histological cryostat sections. We focused our analysis on the mesenteric tissue within the gastroderm. After 30 h post infection at 27°C, anemones presented bacterial clusters in the mesenterial filaments (**Figure 4**). The bacteria were scattered in the damaged tissue, as shown by the 3D view, and were arranged in a round shape. This was in contrast to what we saw at 31°C, where we failed to see bacterial clusters (**Figure 4**).

Our study revealed that bacteria exhibited a clustered arrangement within the anemone’s mesenterial filaments after 24 h of infection at 27°C, while at 31°C, the bacteria were scattered, indicating temperature-dependent variations in bacterial spread. We hypothesized that this bacterial clustering was similar to the phagocytosis observed in vertebrates during infection.

### *V. parahaemolyticus* are mainly located in the phagocytic zone of mesenterial filaments of *E. pallida*

**Figure 5:**
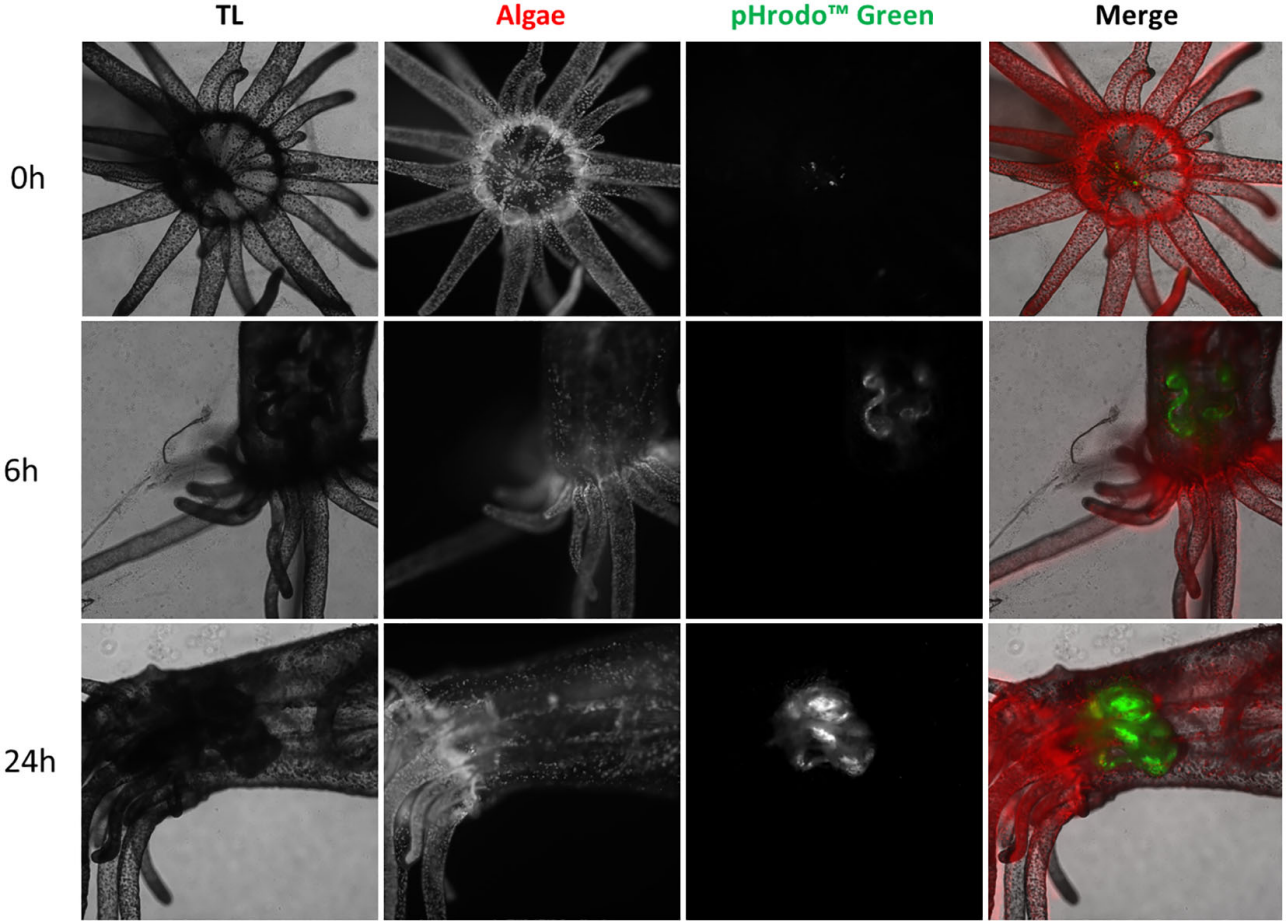
Confocal microscopy of live *E. pallida* inoculated with pHrodo Green Zymosan (in green) immediately after treatment (0 h); or 6 and 24 h post treatment. Autofluorescence of algal symbionts (algae) appears in red. TL: transmitted light.

To localize the phagocytic cells, we use pHrodo™ green zymosan, which highlights phagocytic activity in anemones (21). pHrodo™ Green Zymosan emits fluorescence during phagocytosis, allowing direct observation and tracking of particle uptake by phagocytic cells, providing real-time insight into the dynamic phagocytosis process. We inoculated the particles directly into the FSW medium. Autofluorescence of the symbiont algae was used to visualize all structures of the anemones. After 6 h of inoculation, Zymosan particles’ fluorescence activity was clearly visible inside the mesenterial filaments. This activity increased and persisted after 24 h (**Figure 5**).

To further differentiate the phagocytic and digestive zones of the mesenteries, we used Red DQ™ BSA, a proteinase substrate that highlights the digestion process. Since this probe fluoresces at a similar wavelength of the highly autofluorescent symbiont algae, aposymbiotic anemones (deprived of its algae) were used for these experiments. We inoculated the Red DQ™ BSA and the pHrodo™ Green Zymosan directly into the FSW medium. The digestion of the Red DQ™ BSA was scattered in the whole organism (**Supplementary Figure 1**).

For phagocytic cell localization, we co-inoculated Vp-GFP and Red DQ™ BSA in the whole animal. The bacteria were mainly found engulfed in the mesenterial filaments (**Supplementary Figure 1**). After 24 h, the mucus ring produced by *E. pallida* trapped bacteria and masked the low internal fluorescence signature (**Supplementary Figure 1**). Therefore, we dissected the anemones and focused our observations on the mesenterial filaments, which show differential localization of Vp-GFP and the Red DQ™ BSA even after 24 h in FSW (**Supplementary Figure 2**).

Throughout the anemone, the bacteria were located in the mesenterial filaments. The dissection allowed us to identify different zones for the digestive process and the phagocytosis. We therefore focused on the identification of different populations of *E. pallida* cells.

### *E. pallida* shows a diversity of autofluorescent cell populations

**Figure 6:**
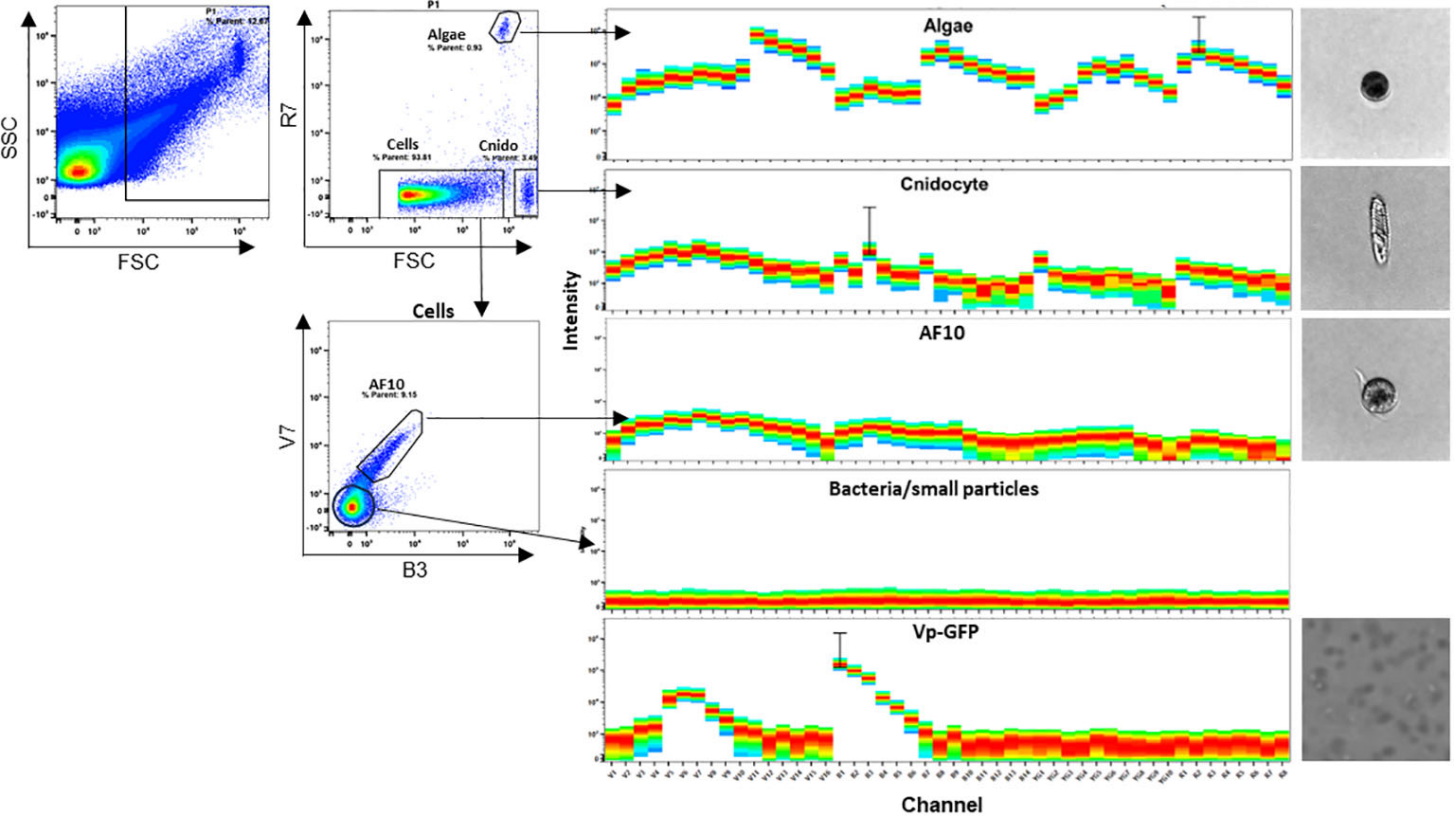
*E. pallida* cell populations identified by spectral flow cytometry. Four spectra were identified as Algae, Cnidocyte, AF10, and bacteria/small particles and were imaged. FSC corresponds to forward scatter, SSC to side scatter, R to red laser, B to blue laser, and V to violet laser.

**Figure 7:**
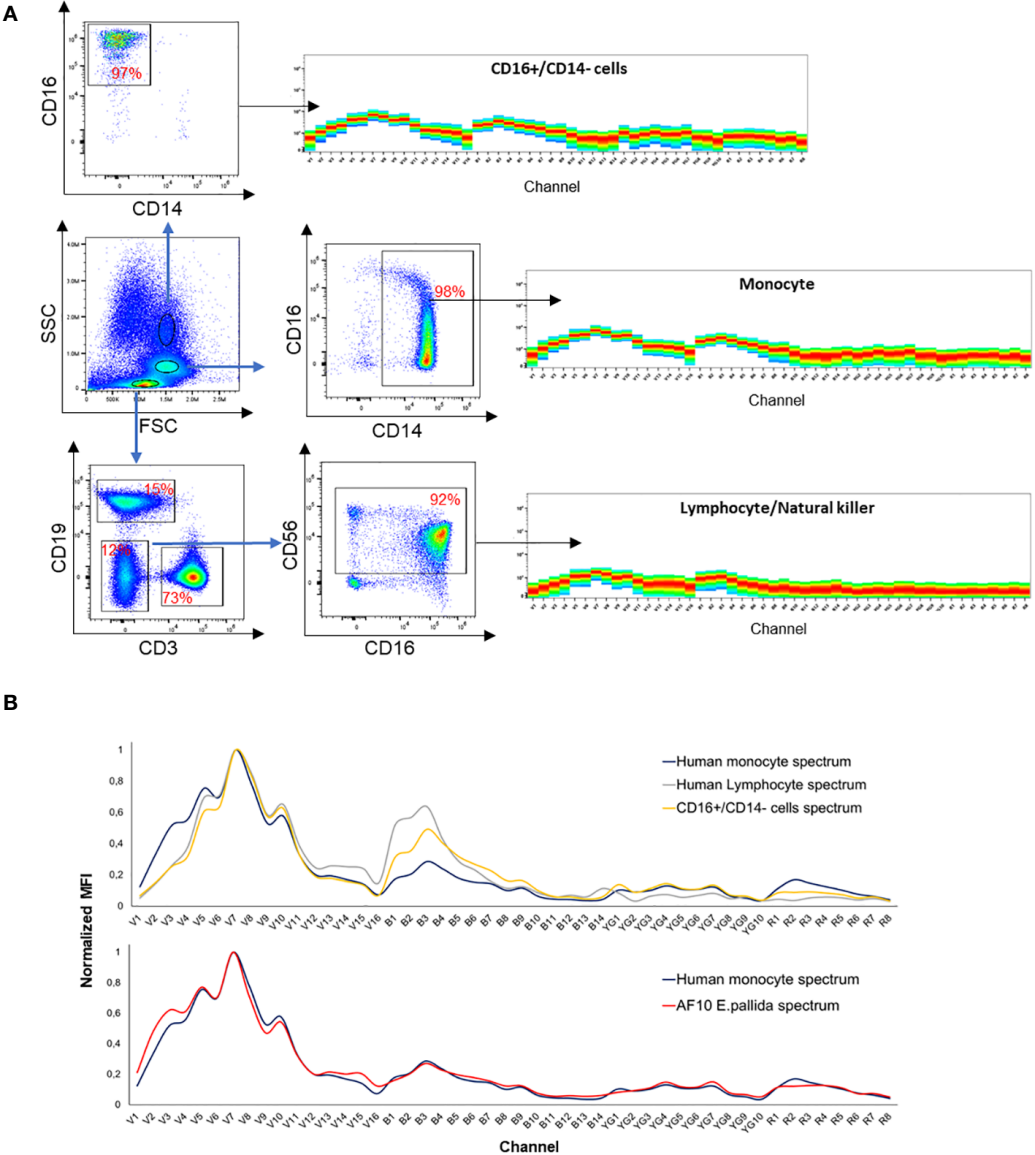
**(A)** Spectra identification of human white blood cells. **(B)** Spectra overlay between human monocyte and AF10 population spectrum and spectra overlay between different human white blood cells.

Here, we used spectral flow cytometry (SFC) to identify different cell types in *E. pallida*. We tracked three parameters: cell size, granularity, and autofluorescence. This allows us to assign spectra to different populations that have different sizes and granularities (**Figure 6**). Three separate populations and their spectra were identified according to the ‘‘similarity index’’ provided by the software Spectroflow from Cytek. Each population was sorted and identified morphologically under the microscope as Cnidocytes, algae, or AF10 population. To quantify these populations in different tissues of *E. pallida*, the animals were dissected into four tissues (tentacles, body, acontia, and mesenterial filaments). Cells were extracted, dissociated, and populations were quantified according to their spectral profiles. Cnidocytes and algae were present in all tissues; however, their quantities varied depending on the tissue (data not shown). In contrast, AF10 population was found to have equal distribution in different tissues and the content was not affected by the temperature (**Supplementary Figure 3**). Because of the high genetic homology in innate immunity between *E. pallida* and humans, we attempted to identify the immune cells of *E. pallida* by comparing their autofluorescence with that of human white blood cells. This analysis revealed three distinct autofluorescence spectra corresponding to specific cell populations: lymphocytes/natural killer cells (CD3+ and CD56+ cells), monocytes (CD14+ cells), and polymorphonuclear cells with macrophages (CD16+/CD14− cells), as shown in **Figure 7**. When we compared these spectra with those identified in *E. pallida*, we found that the AF10 spectrum had a remarkable 99% similarity to the monocyte and macrophage populations. In contrast, the similarity with lymphocytes/NK cells was lower, with a similarity index of 0.96.

### Identification of an “amebocyte-like cell” population with phagocytosis properties

**Figure 8:**
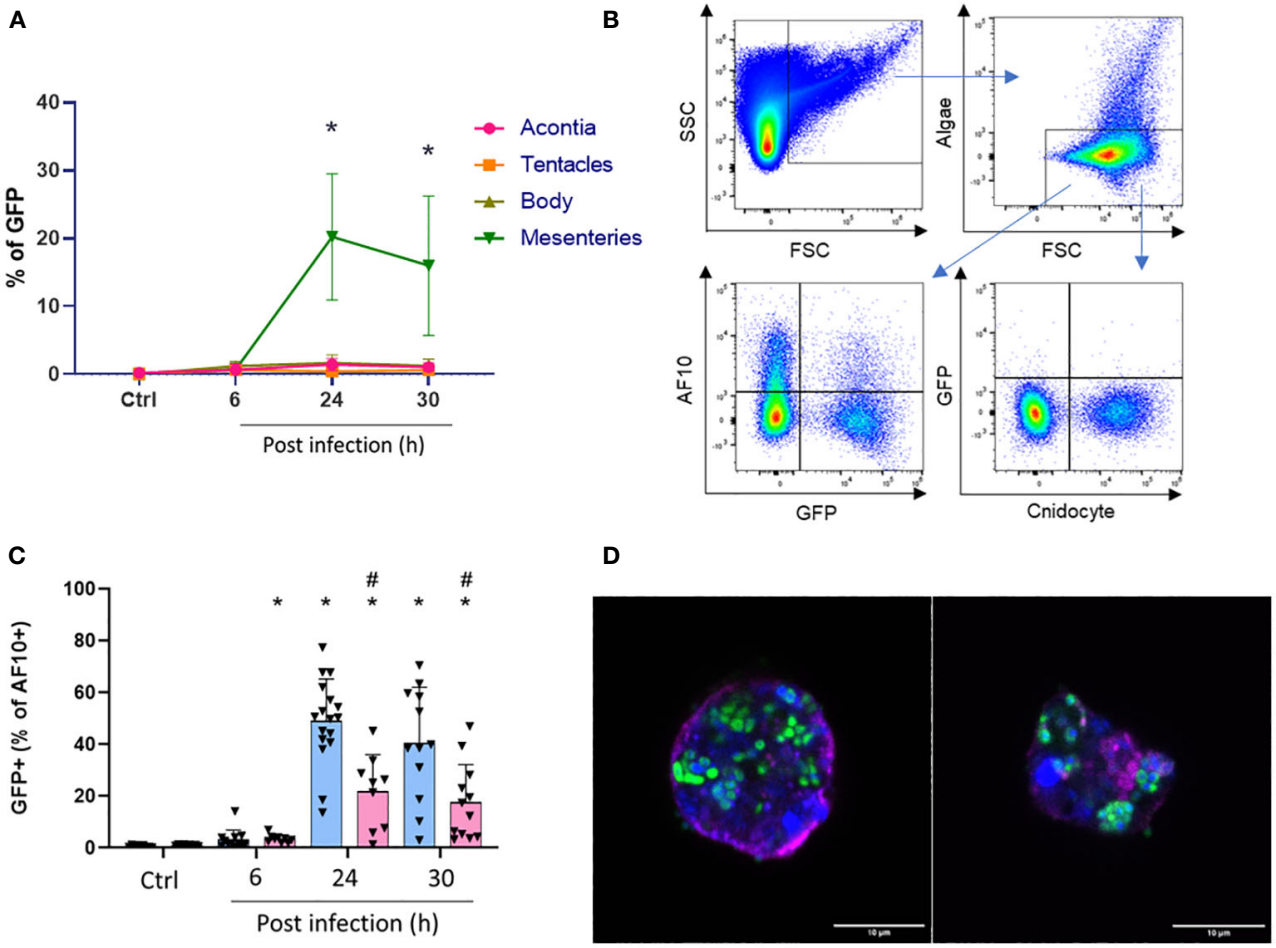
**(A)** Quantification of GFP population in each tissue over infection. *Significant difference compared to control at 27°C. **(B)** Gating strategy for the quantification of double-positive population (with GFP). **(C)** Calculated ratio of AF10+/GFP+ from total cells expressed as a percent of controls or infected anemones after 6, 24, and 30 h at 27°C (blue) or 31°C (red). ^#^Significant difference between temperatures for the same time point. *Significant difference compared to control at the same temperature. **(D)** Sorted cells of AF10+/GFP+ population. Nuclei are labeled with DAPI (blue); membranes with BioTracker655 and Vp-GFP are in green. Scale bar = 10 µm.

To investigate if the AF10 population shares phagocytosis properties with “amebocyte-like cells”, we focused on the localization of Vp-GFP in *E. pallida*-infected animals at 27°C and 31°C over the period of infection. Animals were dissected and we used the GFP spectrum to quantify Vp in different tissues. The mesenterial filaments was found as the main tissue containing Vp-GFP (**Figure 8A**; **Supplementary Figure 4**). Then, we localized the Vp-GFP in different cell populations (**Figure 8B**). Cnidocytes and algae were not found undergoing infection (**Supplementary Figures 5**–**7**). AF10 cells extracted from mesenterial filaments expressed the highest level of GFP, suggesting the presence of Vp within the cells (**Supplementary Figure 4**). Moreover, at late time post infection (24 and 30 h), we observed a significant decrease in the AF10+/GFP+ cell population at 31°C compared to 27°C (**Figure 8C**; **Supplementary Figure 8**). We then sorted the AF10+/GFP+ population and examined the localization of vesicle-containing bacteria under a confocal microscope.

We confirmed that the observed structures of sorted cells contained a nucleus, an undamaged membrane, and bacteria clusters (Vp-GFP) suggesting that, indeed, they were phagocytic cells (**Figure 8D**). The size of these cells was approximately 10 µm and similar to the vesicle-containing bacteria observed in the histological section of mesenterial filaments after 24 h of infection at 27°C (**Supplementary Figures 9**–**11**). However, in anemone mesenteries infected at 31°C, we did not observe clustered bacteria and they were scattered throughout the tissue. Spectral flow cytometry confirmed that these cells containing bacterial clusters were low represented at 31°C (**Figure 5**; **Supplementary Figure 8**).

## Discussion

*Exaiptasia pallida* is a valuable model for studying host–pathogen interactions and underlying immune responses. Understanding how the immune system of *E. pallida* responds to infection with *V. parahaemolyticus* may provide insights into the pathogenesis and virulence mechanisms of this bacterium in marine environments. In addition, studying the effect of temperature on the response is critical, as *V. parahaemolyticus* infections are often associated with warmer water temperatures (3, 29, 30) and can also be pathogenic to humans (28).

Recently, the IPCC released results showing that the mean temperature of the tropical ocean is rising faster than expected and this rise of SWT has an impact on the virulence of bacteria. We have recently demonstrated that at 31°C, *V. parahaemolyticus* overexpressed adhesins and pili when compared to bacteria maintained at 27°C (8). These factors are implicated in adhesion and biofilm formation on the biotic surface, thus rendering the bacteria more stressful for the host.

The rise in SWT also impacts the health of marine organisms. The anemone *E. pallida* is known to usually live in the tropical sea between 27°C and 29°C without any stress. With a low temperature variation at 31°C, the anemones presented a signal of stress but recovered to a normal appearance after a period. However, at 33°C and 35°C, the anemones were severely stressed and unable to recover. This study shows the possible acclimatization of *E. pallida* facing a limited rise in temperature (31).

According to the literature, increasing SWTs have significant effects on anemones, affecting their physiological and ecological dynamics. For example, Black et al. (1995) (31) showed that *E. pallida* produces higher levels of HSP68 and HSP72 after short-term thermal stress. HSPs are involved in controlling the folding, assembly, and transport of cell proteins under normal and stress conditions in a large panel of species (32). In our study, anemones were transferred from 27°C to 31°C for at least 2 weeks, to overcome the possible incidence of the short thermal shock during infection. We verified that after 2 weeks of acclimatization, HSP gene expressions return to the level of control cells (data not shown). In vertebrates, heat shock proteins are also implicated in response to the infection. In infected anemones at 27°C, we observed a significant overexpression of HSP70 at 6 h post infection. This confirms our transcriptomic analysis performed on *E. pallida* infected at 27°C showing that Vp induces increased expression of HSP70 during infection with Vp (18). In infected anemone at 31°C, HSP40 and HSP70 are significantly overexpressed at 24 and 30 h post infection. This suggests that temperature and *V. parahaemolyticus* infection had a synergetic effect on regulating the heat shock response in *E. pallida.*

To visualize the evolution of the infectious process, we used the clearing protocol ‘‘AnemoClear’’. Immediately after infection, bacteria adhered to the external tissue and was localized inside the animal 24 h post infection at 27°C, mainly localized in the mesenterial filaments. According to literature, mesenterial filaments are known for being a zone of phagocytosis and digestion (33, 34). Furthermore, anemones’ mesenterial filaments showed inflamed mesenteric structures after 30 h of infection at 27°C, which look like inflamed ganglion structures found in humans (**Figure 3**).

To exclude the degradation of bacteria by the digestion process, we performed experiments to localize the phagocytic and digestive zones. For this purpose, we dissected anemones to observe more precisely the mesenterial filaments. No colocalization was found between Vp-GFP and DQ™ BSA. However, the pHrodo™ green zymosan particles in anemones showed a similar localization to the Vp-GFP. The phagocytic assays confirm the localization of phagocytic engulfment processes that are different from the digestive one.

According to the literature, the phagocytosis process is conserved in cnidarians (20, 21, 25). Phagocytosis is the cellular defense mechanism used to eliminate antigens derived from dysregulated or damaged cells and microbial pathogens. Phagocytosis is therefore a pillar of innate immunity, whereby foreign particles are engulfed and degraded in lysolitic vesicles. In hexacorallia, phagocytic mechanisms are poorly understood, though putative anthozoan phagocytic cells (amebocytes) have been identified histologically.

Having established the presence of the phagocytosis process, the next crucial step in our research was the detailed examination of the cells involved. This investigation was essential to accurately identify the specific cell type involved in the phagocytosis process.

Cytometry is a powerful technique for analyzing and quantifying the phenotype of cells and particles. It is widely used in various fields, including biology, medicine, and immunology (35). Using flow cytometry has become a useful tool for identification of different cell types in marine organisms. As specific markers and antibodies are lacking in the field of marine biology, we achieved this task thanks to side scatter parameters. A similar technique has been used for the identification of phytoplankton or hemocyte populations in the mussel *Mytilus galloprovincialis* (36, 37). In rare cases, markers such as the propidium iodide was used to quantify the viability of hemocytes isolated from *Crassostrea virginica* (38). Rosental et al. (2017) (39) optimized a large panel of non-species-specific markers for studying the cellular role of stress response as well as for immunological studies in corals and other cnidarians.

Spectral flow cytometry could help overcome the problem of lacking markers and specific antibodies. The advantage of using spectral cytometry is that it allows us to characterize cell populations by autofluorescence. Natural autofluorescence is a widespread phenomenon observed in different types of marine organisms such as microalgae, oceanic fungi, and planktonic ciliates (40–42).

This unique approach allowed us to characterize different populations of *E. pallida* as cnidocyte, algae, and the AF10 named “amebocyte-like cell” populations. In *E. pallida*, the AF10 population was found in all tissues (i.e., Ectoderm, Mesoglea, and Endoderm), as amebocyte cells according to Berzins et al. (2021) (20). According to the similarity index provided by Cytek, the “amebocytes-like cell” had a similarity of 0.99 with human monocytes/macrophages and shared some common morphological aspects.

Amebocyte cells have been shown to play a role in phagocytic and bactericidal capability in cnidarians including corals, anemones, and gorgones (20, 22, 23). Furthermore, it has been reported that phagocytosis in *E. pallida* is a temperature-dependent process. Moreover, although temperature increases immune cell activity during heat stress in *E. pallida* after 12 h (25), prolonged exposure can decrease this activity in other marine species (43, 44).

During infection, the cnidocyte and algae populations were not GFP positive whereas “amebocytes-like cell” were positive for GFP (AF10+/GFP+). Those cells were mainly found in the mesenterial filaments 24 h post infection at 27°C and confocal microscopy showed that these cells measuring 10 µm contained a nucleus and bacteria-filled vesicles. Interestingly, there was a low number of AF10+/GFP+ cells present in anemones infected at 31°C and bacteria were found scattered in mesenteries. One explanation could be that the anemone has reached its thermal limit response. Ben-Haim et al. (2003) (45) have shown that in case of *P. damicornis* infected by *V. coralliilyticus*, an increase of sea temperature by 3°C can limit the magnitude of immune responses of this marine organism, inducing bleaching and even lysis of the coral.

In conclusion, we have demonstrated that AF10 cells have been shown to phagocytose *V. parahaemolyticus* in a temperature-dependent process. We identified AF10 as “amebocyte-like cells” in *E. pallida* based on their autofluorescence spectrum, which are similar to human monocytes/macrophages spectrum. Beyond the effect of temperature on the phagocytic activity, our approach demonstrates that spectral flow cytometry is a useful tool for studying marine organisms. Single-cell analyses remain to be performed to characterize the ‘‘amebocytes-like cell’’ and understand their precise function during infection.

## Data availability statement

The original contributions presented in the study are included in the article/**Supplementary Material**. Further inquiries can be directed to the corresponding author.

## Ethics statement

The studies involving humans were approved by French national agency for blood collection (79-52-NQ). The studies were conducted in accordance with the local legislation and institutional requirements. The participants provided their written informed consent to participate in this study. The manuscript presents research on animals that do not require ethical approval for their study.

## Author contributions

MB: Data curation, Formal Analysis, Investigation, Methodology, Resources, Software, Validation, Visualization, Writing – original draft. FL: Formal Analysis, Investigation, Methodology, Validation, Visualization, Writing – original draft. DC: Conceptualization, Funding acquisition, Project administration, Supervision, Validation, Writing – original draft.

## Supplementary material

Supplementary Figure 1 Confocal microscopy of live whole *E. pallida* controls or infected by Vp-GFP (in green) and simultaneously inoculated with DQ BSA (red) after 6 and 24 hours at 27°C. TL: Transmitted light.

Supplementary Figure 2 Confocal microscopy of fixed mesenterial filaments of *E. pallida* controls or infected by Vp-GFP (in green) and simultaneously inoculated with DQ BSA (red) at 6 or 24 hours at 27°C. TL: Transmitted light.

Supplementary Figure 3 Quantification of the AF10 population in the different tissues at 27 or 31°C in controls **(A)** or infected anemones: **(B)** Acontia, **(C)** Tentacles, **(D)** Body and **(E)** Mesenterial filaments. Experimentations were done at 27°C (in blue) or 31°C (in red). Sampling was done after 6, 24 and 30 hours of infections (n=9).

Supplementary Figure 4 GFP+ quantification in controls or infected anemones organs: **(A)** Acontia, **(B)** Tentacles, **(C)** Body and **(D)** Mesenterial filaments. Experimentations were done at 27°C (in blue) or 31°C (in red). Sampling was done after 6, 24 and 30 hours of infections (n=9).

Supplementary Figure 5 Cnidocytes quantification in controls or infected anemones organs: **(A)** Acontia, **(B)** Tentacles, **(C)** Body and **(D)** Mesenterial filaments. Experimentations were done at 27°C (in blue) or 31°C (in red). Sampling was done after 6, 24 and 30 hours of infections (n=9).

Supplementary Figure 6 Cnido+/GFP+ quantification in controls or infected anemones organs: **(A)** Acontia, **(B)** Tentacles, **(C)** Body and **(D)** Mesenterial filaments. Experimentations were done at 27°C (in blue) or 31°C (in red). Sampling was done after 6, 24 and 30 hours of infections (n=9).

Supplementary Figure 7 Algae quantification in controls or infected anemones organs: **(A)** Acontia, **(B)** Tentacles, **(C)** Body and **(D)** Mesenterial filaments. Experimentations were done at 27°C (in blue) or 31°C (in red). Sampling was done after 6, 24 and 30 hours of infections (n=9).

Supplementary Figure 8 AF10+/GFP+ quantification in controls or infected anemones organs: **(A)** Acontia, **(B)** Tentacles, **(C)** Body and **(D)** Mesenterial filaments. Experimentations were done at 27°C (in blue) or 31°C (in red). Sampling was done after 6, 24 and 30 hours of infections (n=9).

Supplementary Figure 9 Confocal microscopy of histological section of infected *E. pallida* by Vp-GFP at 27°C. Zoom focuses on cells with filled-bacteria vesicles after 24 hours of infection. Nuclei were labelled by DAPI (blue); Mucus by WGA (red); Vp express constitutively GFP (green) and membrane by BioTracker655.

Supplementary Figure 10 Confocal microscopy of histological section of infected *E. pallida* by Vp-GFP at 27°C. Zoom focuses on cells with filled-bacteria vesicles after 24 hours of infection. Nuclei were labelled by DAPI (blue); Mucus by WGA (red); Vp express constitutively GFP (green) and membrane by BioTracker655.

Supplementary Figure 11 Z-stack of the observation in **Figure Supplementary 9** showing different sections of the filled-bacteria vesicles. Scale bar = 10 µm.
