# Supplementary material for: Impact of rising seawater temperature on a phagocytic cell population during V. parahaemolyticus infection in the sea anemone E. pallida
Source: Front Immunol. 2023 Nov 22;14:1292410. doi: 10.3389/fimmu.2023.1292410 (PMC10703433; doi:10.3389/fimmu.2023.1292410)
Supplement: Supplementary file 1 [file Table_1.docx]

| Table 1 | Primers and sequences | |  |
| --- | --- | --- | --- |
|  |  |  |  |
| Target Gene | Direction | Primer sequence (5'-3’) | Reference |
| RPL11 | Forward | TTGGGCCTCTGACAGTACAGTGAACA | (12) |
|  | Reverse | AGCCAAGGTCTTGGAGCAGCTTA |  |
| Hsp40 | Forward | TCGAGATGCAACAACTAACC | This study |
|  | Reverse | CAAAGTGAAAACCAAAACCACC |  |
| Hsp70 | Forward | GACACGAGCGGAACAGATCA | This study |
|  | Reverse | TCCTCCAGCACAGAAGCAAG |  |
