# Supplementary figures and images for: Impact of rising seawater temperature on a phagocytic cell population during V. parahaemolyticus infection in the sea anemone E. pallida
Source: Front Immunol. 2023 Nov 22;14:1292410. doi: 10.3389/fimmu.2023.1292410 (PMC10703433; doi:10.3389/fimmu.2023.1292410)

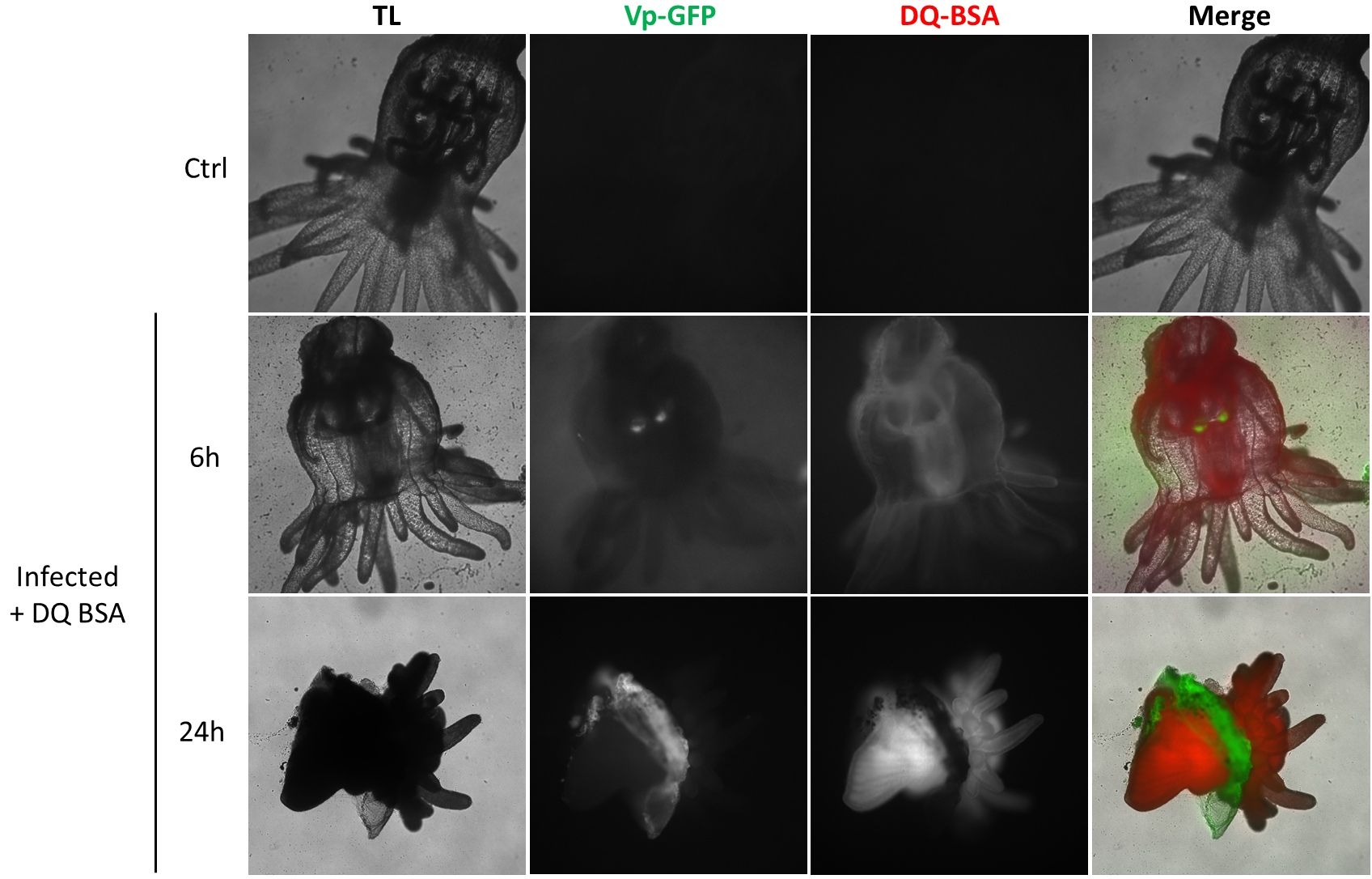

Supplement: Supplementary Figure 1 — Confocal microscopy of live whole E. pallida controls or infected by Vp-GFP (in green) and simultaneously inoculated with DQ BSA (red) after 6 and 24 hours at 27°C. TL: Transmitted light. [file Image_1.tif]

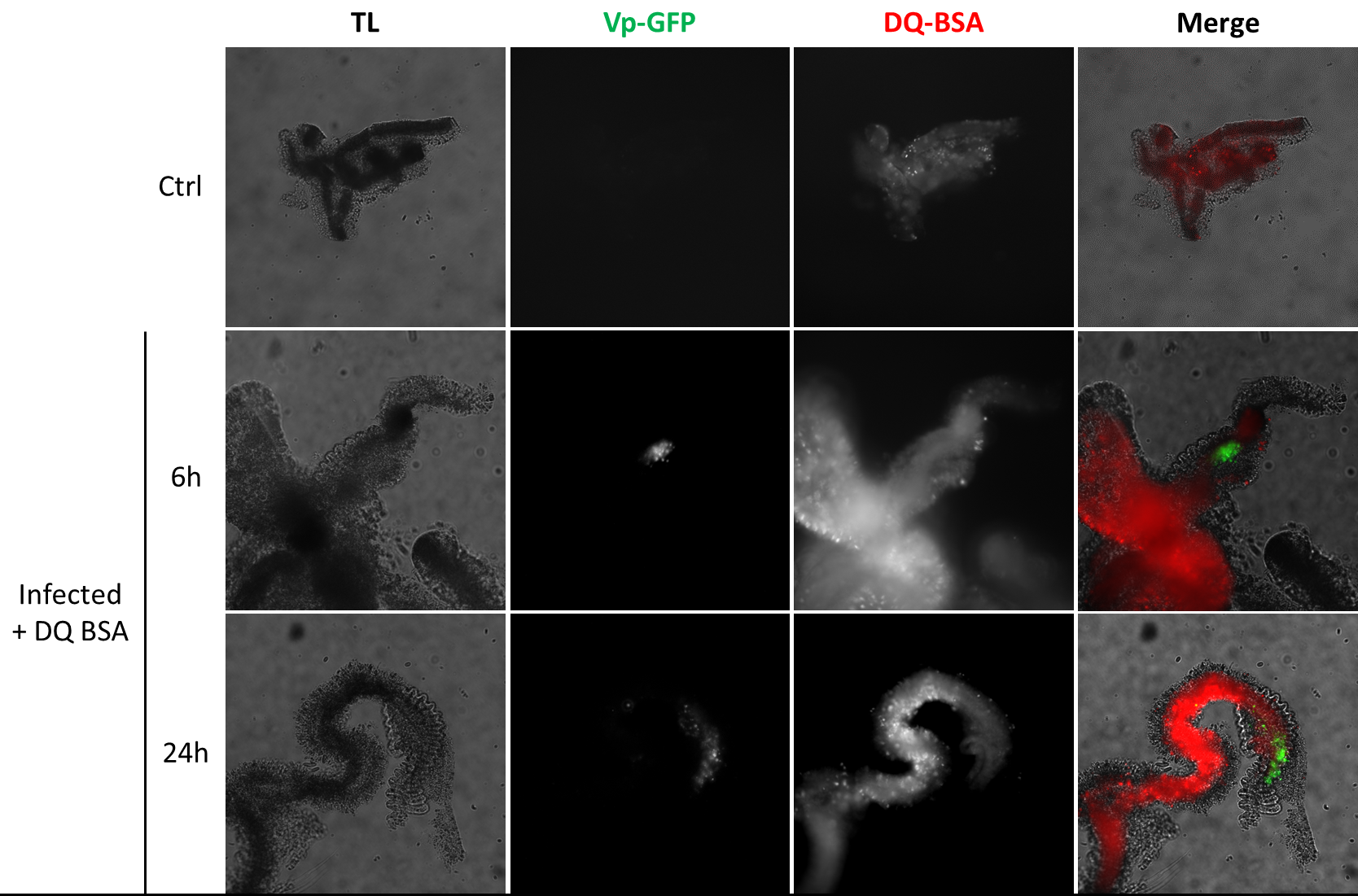

Supplement: Supplementary Figure 2 — Confocal microscopy of fixed mesenterial filaments of E. pallida controls or infected by Vp-GFP (in green) and simultaneously inoculated with DQ BSA (red) at 6 or 24 hours at 27°C. TL: Transmitted light. [file Image_2.tif]

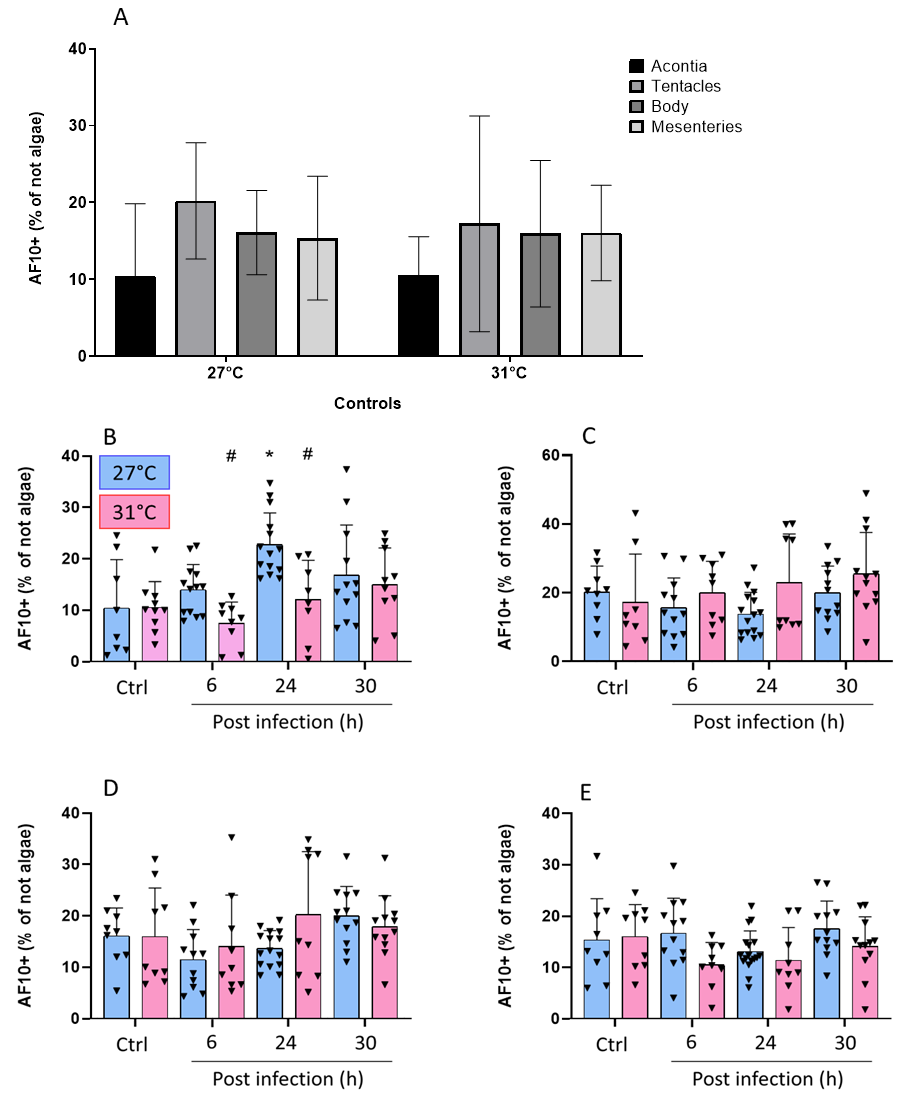

Supplement: Supplementary Figure 3 — Quantification of the AF10 population in the different tissues at 27 or 31°C in controls (A) or infected anemones: (B) Acontia, (C) Tentacles, (D) Body and (E) Mesenterial filaments. Experimentations were done at 27°C (in blue) or 31°C (in red). Sampling was done after 6, 24 and 30 hours of infections (n=9). [file Image_3.tif]

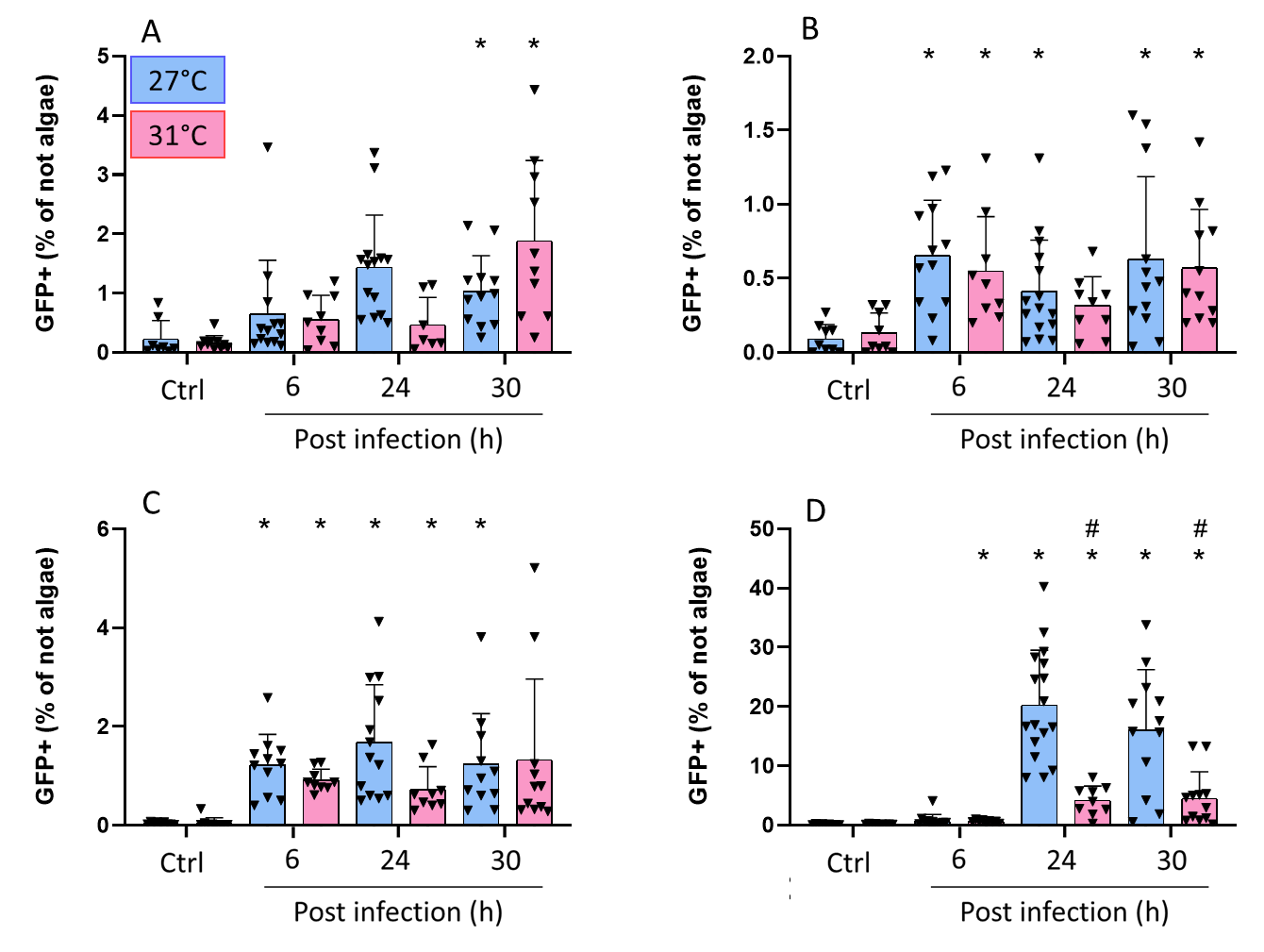

Supplement: Supplementary Figure 4 — GFP+ quantification in controls or infected anemones organs: (A) Acontia, (B) Tentacles, (C) Body and (D) Mesenterial filaments. Experimentations were done at 27°C (in blue) or 31°C (in red). Sampling was done after 6, 24 and 30 hours of infections (n=9). [file Image_4.tif]

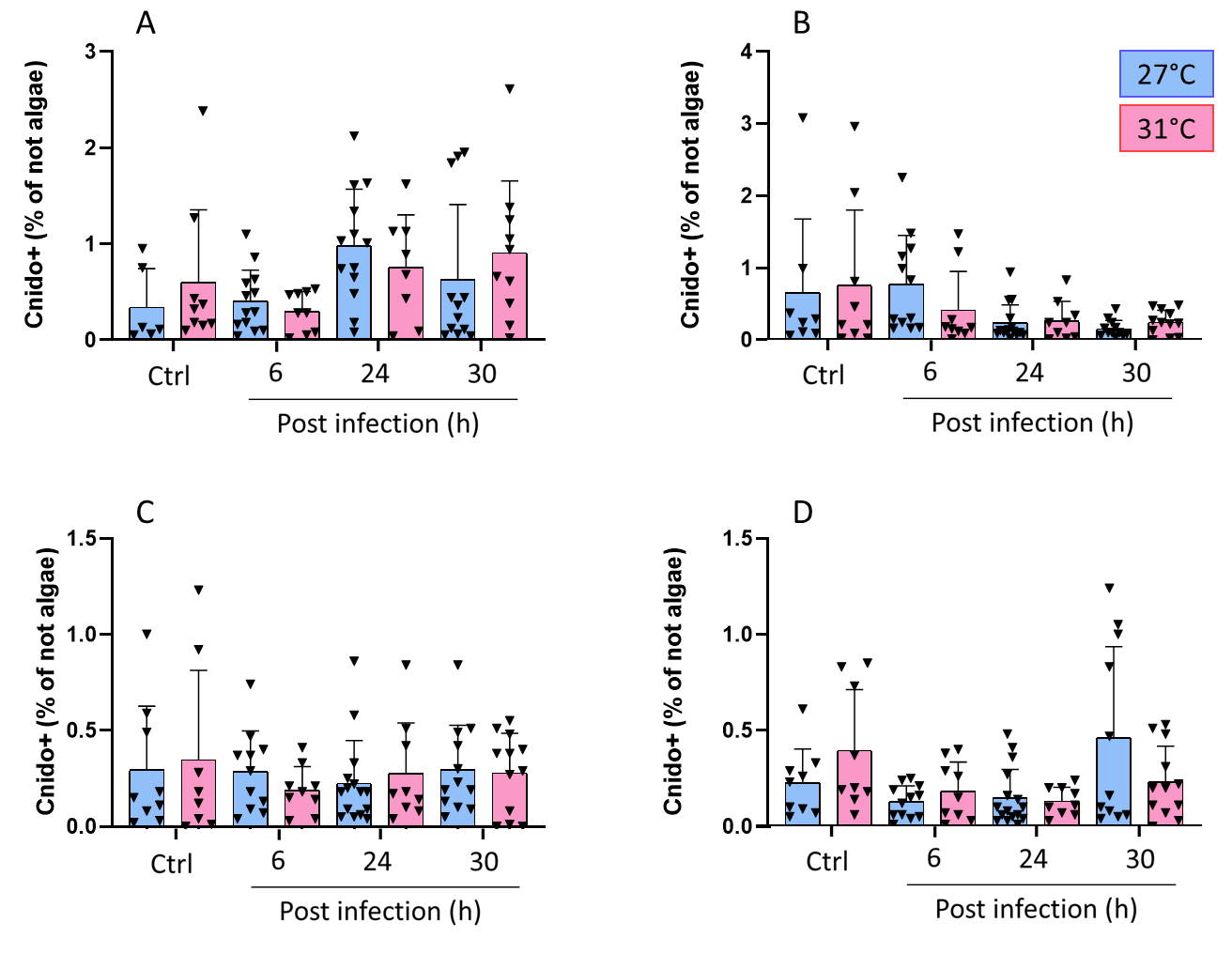

Supplement: Supplementary Figure 5 — Cnidocytes quantification in controls or infected anemones organs: (A) Acontia, (B) Tentacles, (C) Body and (D) Mesenterial filaments. Experimentations were done at 27°C (in blue) or 31°C (in red). Sampling was done after 6, 24 and 30 hours of infections (n=9). [file Image_5.tif]

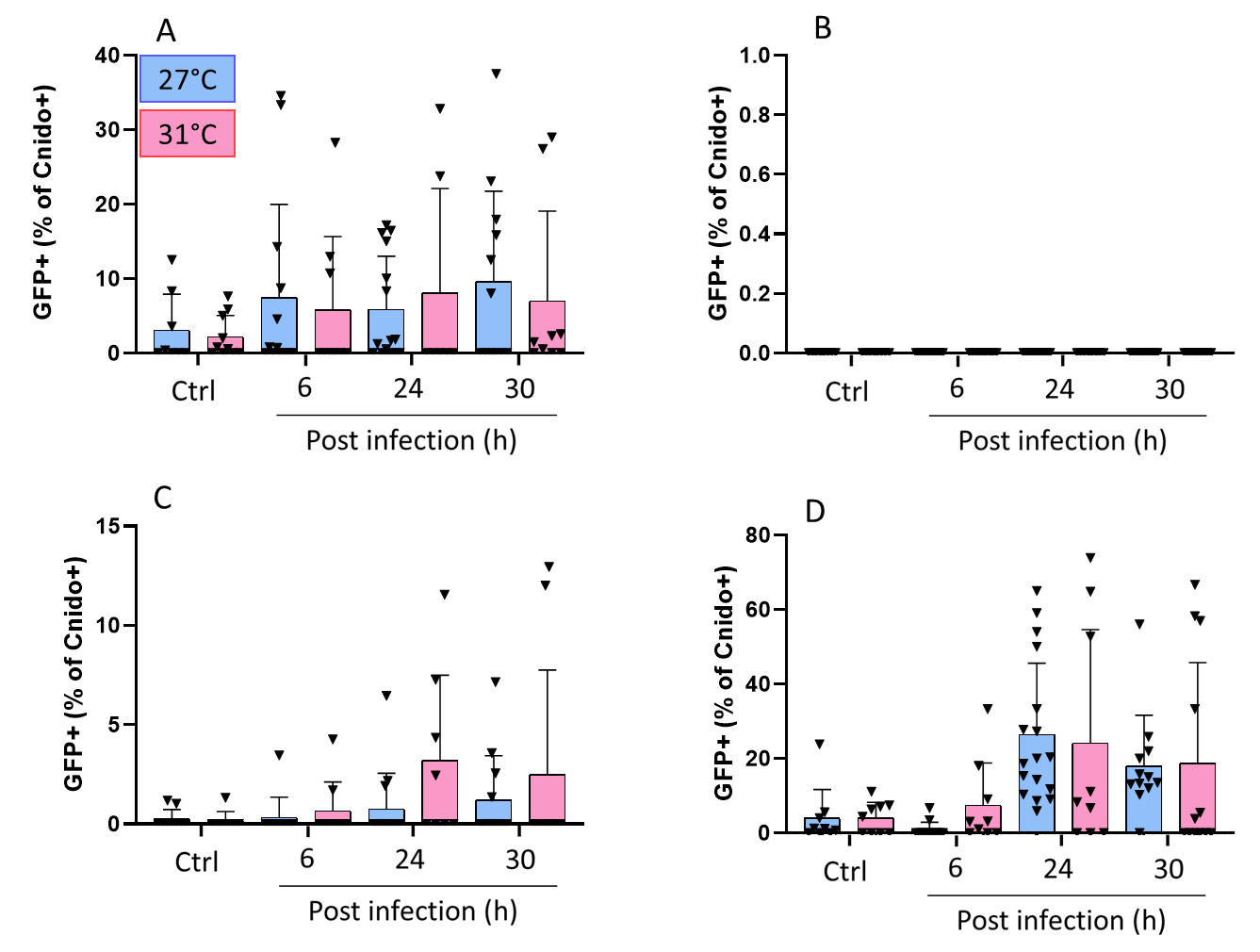

Supplement: Supplementary Figure 6 — Cnido+/GFP+ quantification in controls or infected anemones organs: (A) Acontia, (B) Tentacles, (C) Body and (D) Mesenterial filaments. Experimentations were done at 27°C (in blue) or 31°C (in red). Sampling was done after 6, 24 and 30 hours of infections (n=9). [file Image_6.tif]

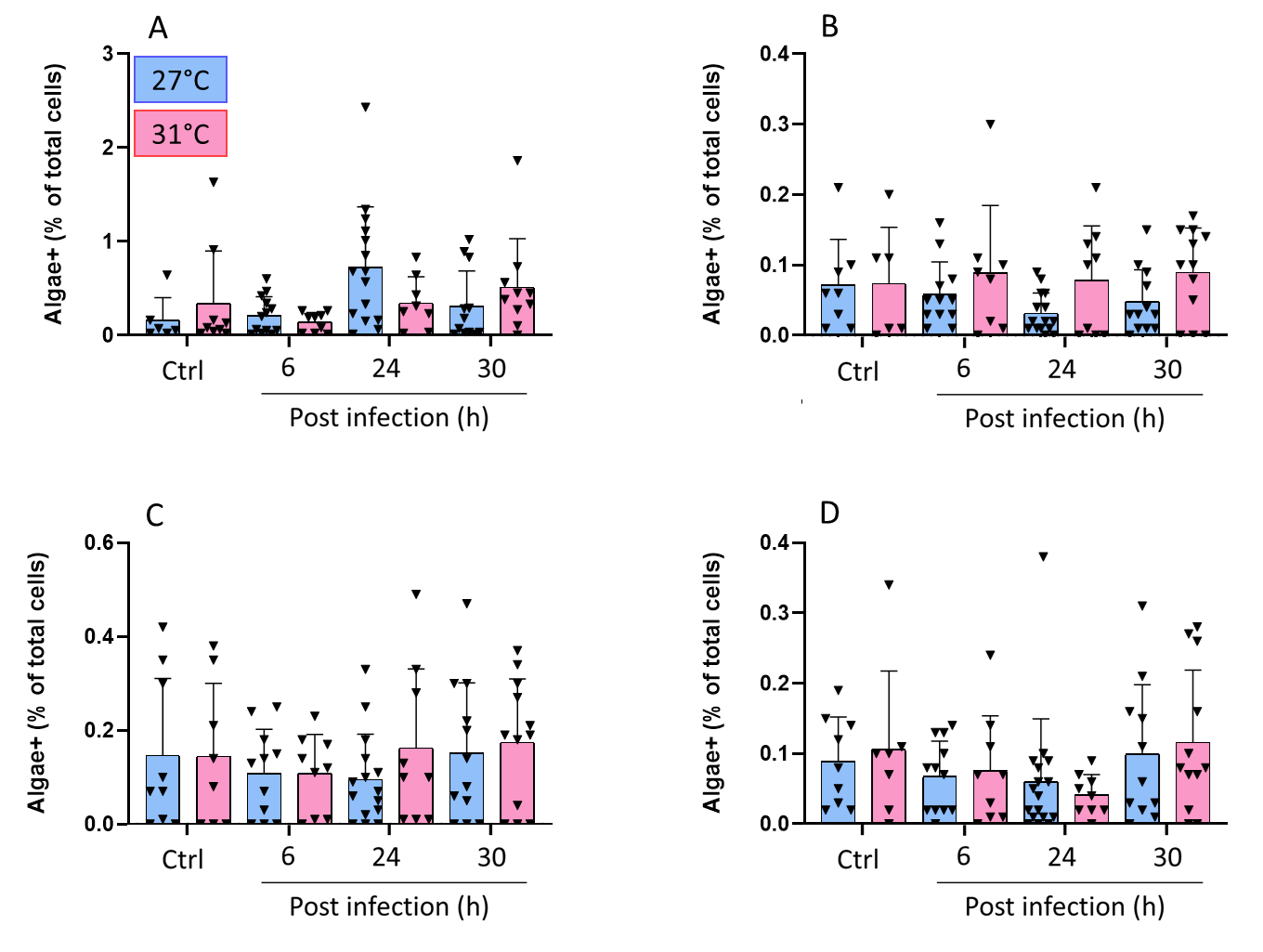

Supplement: Supplementary Figure 7 — Algae quantification in controls or infected anemones organs: (A) Acontia, (B) Tentacles, (C) Body and (D) Mesenterial filaments. Experimentations were done at 27°C (in blue) or 31°C (in red). Sampling was done after 6, 24 and 30 hours of infections (n=9). [file Image_7.tif]

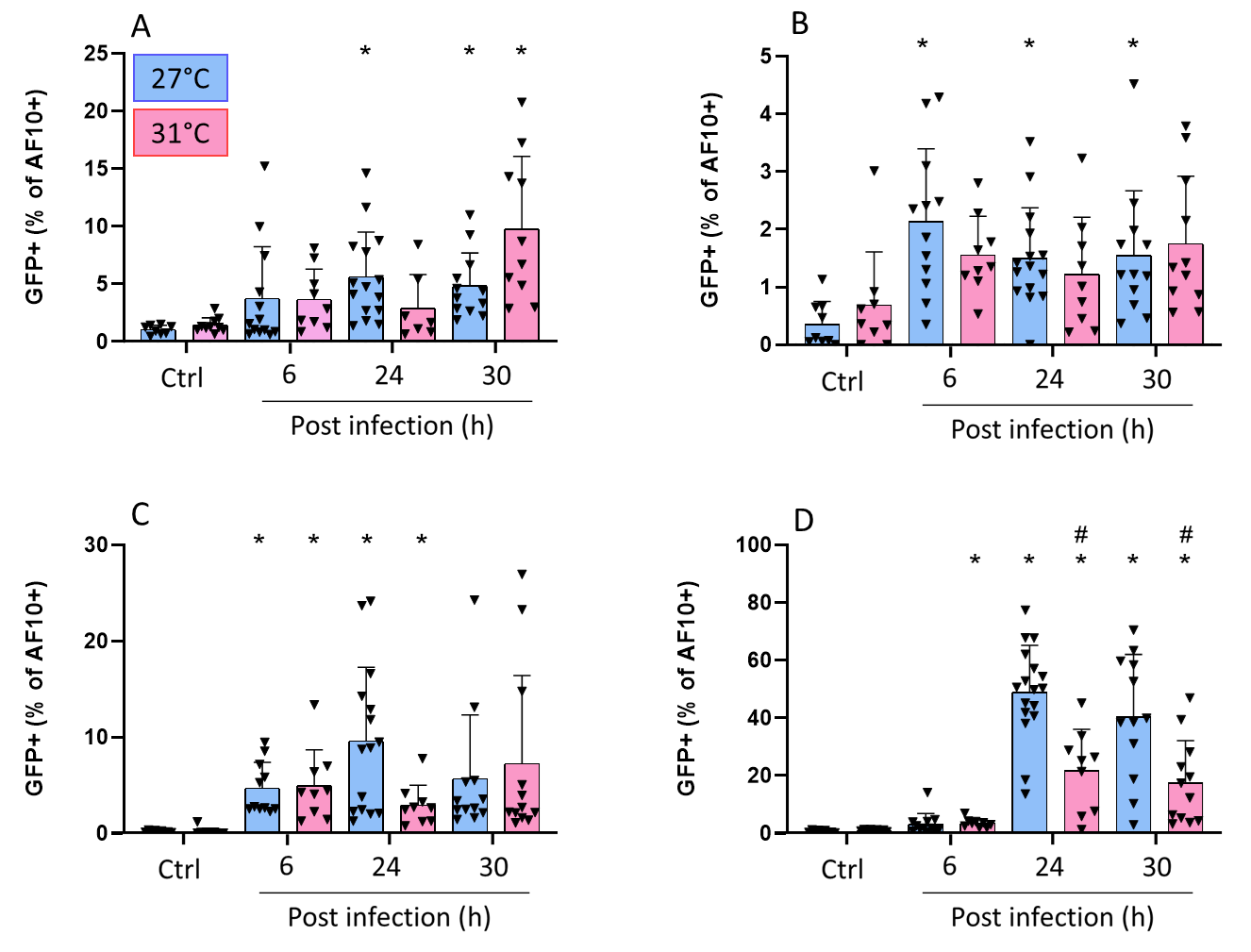

Supplement: Supplementary Figure 8 — AF10+/GFP+ quantification in controls or infected anemones organs: (A) Acontia, (B) Tentacles, (C) Body and (D) Mesenterial filaments. Experimentations were done at 27°C (in blue) or 31°C (in red). Sampling was done after 6, 24 and 30 hours of infections (n=9). [file Image_8.tif]

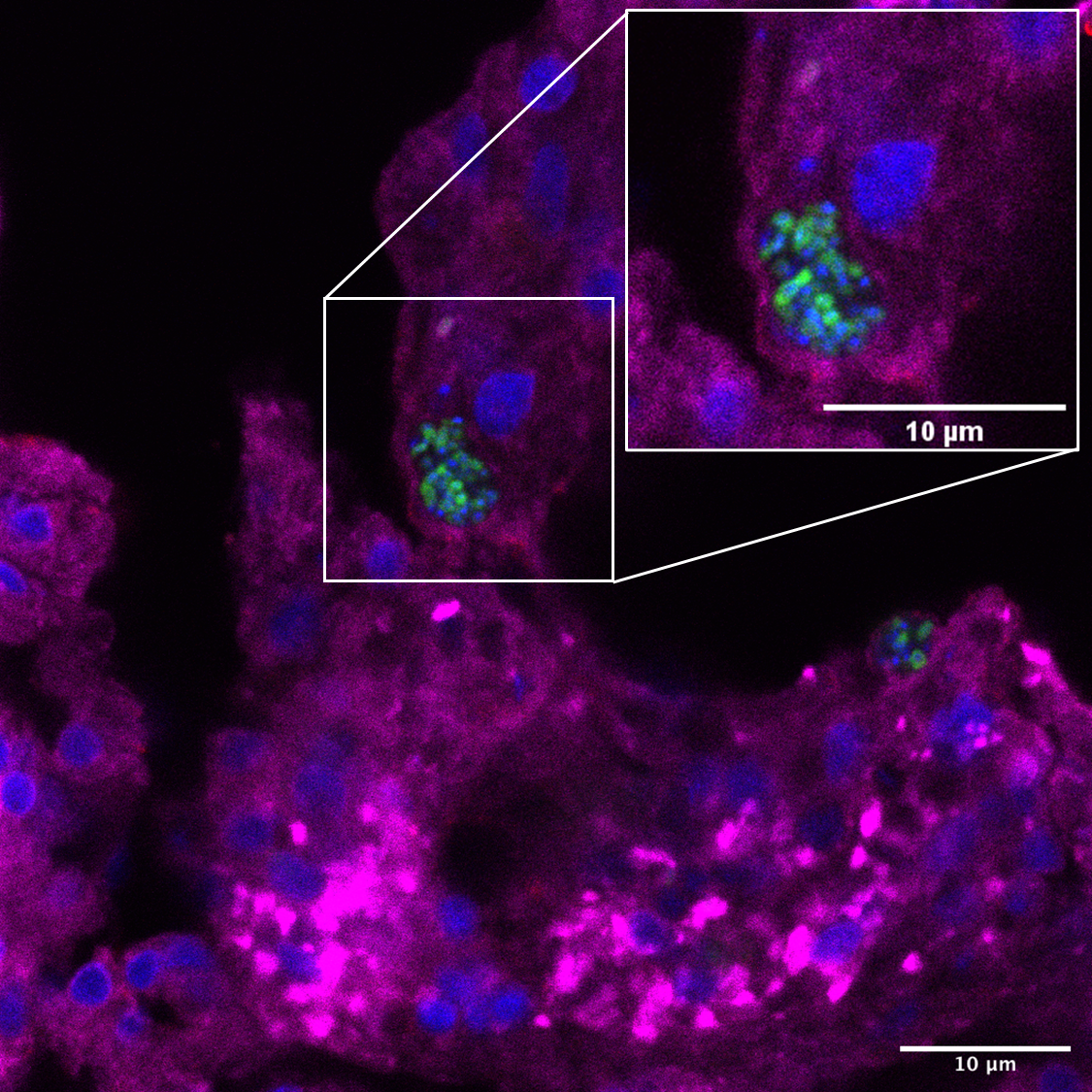

Supplement: Supplementary Figure 9 — Confocal microscopy of histological section of infected E. pallida by Vp-GFP at 27°C. Zoom focuses on cells with filled-bacteria vesicles after 24 hours of infection. Nuclei were labelled by DAPI (blue); Mucus by WGA (red); Vp express constitutively GFP (green) and membrane by BioTracker655. [file Image_9.tif]

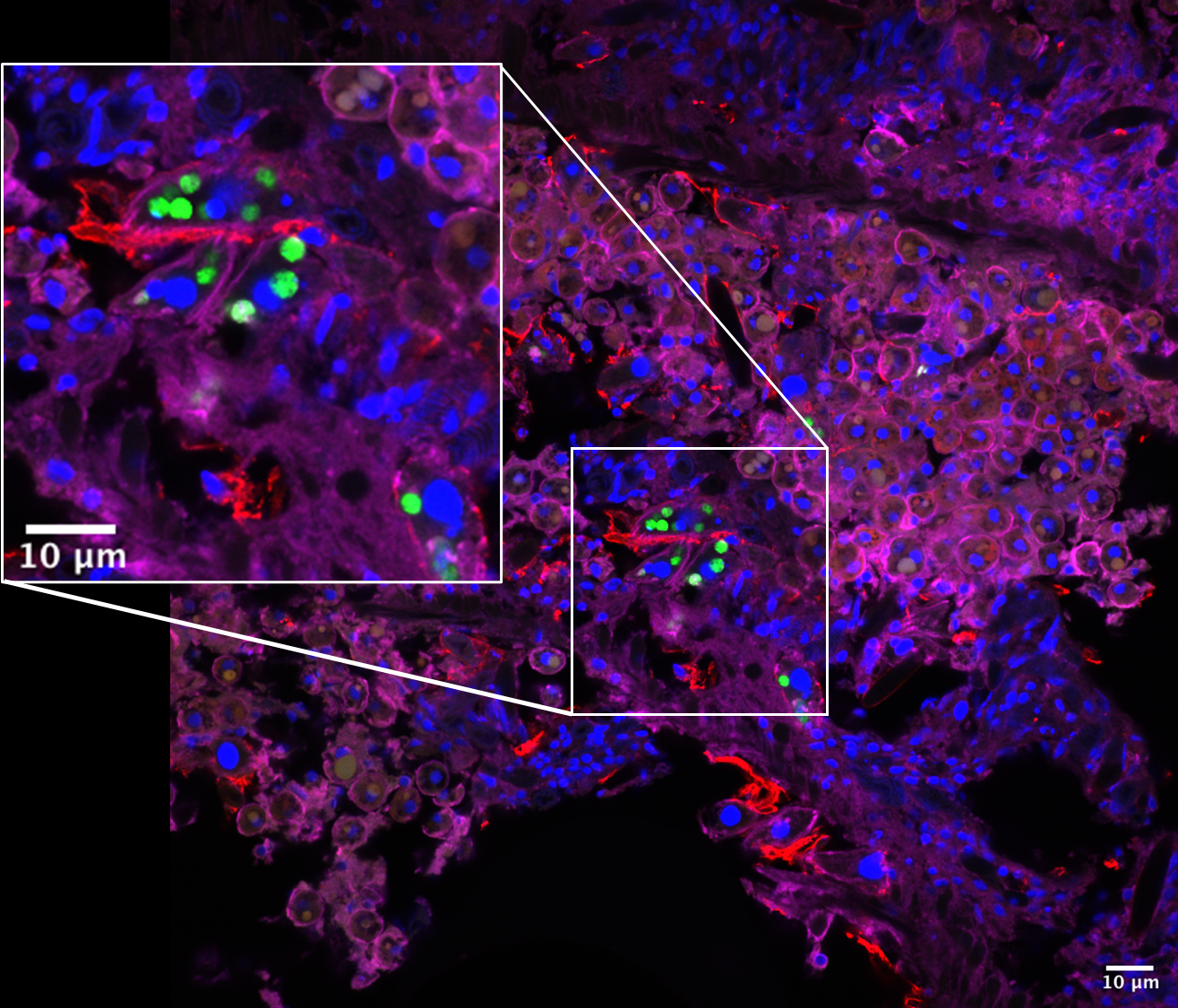

Supplement: Supplementary Figure 10 — Confocal microscopy of histological section of infected E. pallida by Vp-GFP at 27°C. Zoom focuses on cells with filled-bacteria vesicles after 24 hours of infection. Nuclei were labelled by DAPI (blue); Mucus by WGA (red); Vp express constitutively GFP (green) and membrane by BioTracker655. [file Image_10.tif]

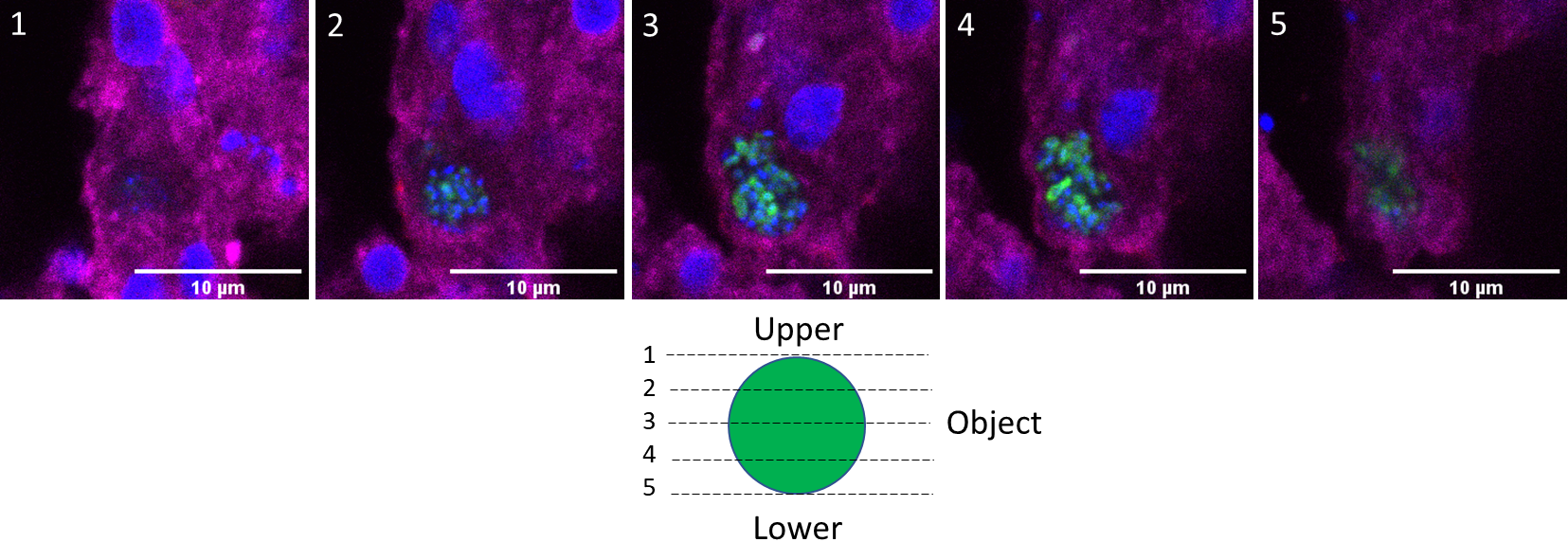

Supplement: Supplementary Figure 11 — Z-stack of the observation in Figure Supplementary 9 showing different sections of the filled-bacteria vesicles. Scale bar = 10 µm. [file Image_11.tif]
